# Genetic diversity and effective population sizes of thirteen Indian cattle breeds

**DOI:** 10.1186/s12711-021-00640-3

**Published:** 2021-06-01

**Authors:** Eva M. Strucken, Netsanet Z. Gebrehiwot, Marimuthu Swaminathan, Sachin Joshi, Mohammad Al Kalaldeh, John P. Gibson

**Affiliations:** 1 Centre for Genetic Analysis and Applications, School of Environmental and Rural Science, University of New England, Armidale, Australia; 2 BAIF Development Research Foundation, Pune, India

## Abstract

**Background:**

The genetic structure of a diverse set of 15 Indian indigenous breeds and non-descript indigenous cattle sampled from eight states was examined, based on 777 k single nucleotide polymorphism (SNP) genotypes obtained on 699 animals, with sample sizes ranging from 17 to 140 animals per breed. To date, this is the largest and most detailed assessment of the genetic diversity of Indian cattle breeds.

**Results:**

Admixture analyses revealed that 109 of the indigenous animals analyzed had more than 1% *Bos taurus* admixture of relatively recent origin. Pure indigenous animals were defined as having more than 99% *Bos indicus* ancestry*.* Assessment of the genetic diversity within and between breeds using principal component analyses, *F* statistics, runs of homozygosity, the genomic relationship matrix, and maximum likelihood clustering based on allele frequencies revealed a low level of genetic diversity among the indigenous breeds compared to that of *Bos taurus* breeds. Correlations of SNP allele frequencies between breeds indicated that the genetic variation among the *Bos indicus* breeds was remarkably low. In addition, the variance in allele frequencies represented less than 1.5% between the Indian indigenous breeds compared to about 40% between *Bos taurus* dairy breeds. Effective population sizes (*N*_*e*_) increased during a period post-domestication, notably for Ongole cattle, and then declined during the last 100 generations. Although we found that most of the identified runs of homozygosity are short in the Indian indigenous breeds, indicating no recent inbreeding, the high *F*_ROH_ coefficients and low *F*_IS_ values point towards small population sizes. Nonetheless, the *N*_*e*_ of the Indian indigenous breeds is currently still larger than that of *Bos taurus* dairy breeds.

**Conclusions:**

The changes in the estimates of effective population size are consistent with domestication from a large native population followed by consolidation into breeds with a more limited population size. The surprisingly low genetic diversity among Indian indigenous cattle breeds might be due to their large *N*_*e*_ since their domestication, which started to decline only 100 generations ago, compared to approximately 250 to 500 generations for *Bos taurus* dairy cattle.

**Supplementary Information:**

The online version contains supplementary material available at 10.1186/s12711-021-00640-3.

## Background

The Indian subcontinent is the center of domestication and current home to most of the world’s *Bos indicus* cattle breeds [1]. *Bos indicus* cattle are renowned for their heat tolerance and adaptation to harsh conditions, making them the predominant type of cattle in tropical countries. Apart from ancient movements of zebu cattle into Africa between 4500 and 700 BCE [2, 3], most of the *Bos indicus* cattle in the rest of the world have been imported from the Indian sub-continent in the past 200 years and may deviate genetically from their original Indian breeds, e.g. Brazilian Nelore or Guzerat [4, 5]. Zebu cattle are believed to originate from the Indian aurochs *Bos primigenius nomadicus* [6]; however, the timing of divergence from taurine cattle is still debated and ranges from 100,000 to 850,000 years ago [7–9]. Archaeological findings that date back to the Neolithic period already indicated that phenotypic differences existed between northern and southern zebu bulls in India, the former with a heavy build and massive horns, and the latter with a light build and a big hump, which suggests that distinct domestication centers occurred in north and south India [10–12].

The number of registered cattle breeds in India has increased steadily over time, with Sukhatme [13] reporting 26 cattle breeds in 1968 and the National Bureau of Animal Genetic Resources (http://www.nbagr.res.in/nbagr.html) listing 50 registered Indian cattle breeds to date. Five of these registered breeds—Sahiwal, Gir, Red Sindhi, Tharparkar, and Deoni—are pure milking breeds, whereas the others are dual purpose or pure draught animals [14, 15]. Only a small percentage of the Indian indigenous breeds are raised as pure breeds and the large majority of cattle used by smallholder farmers are of non-descript breeds [15].

Detailed studies on the genetic diversity of *Bos indicus* cattle are still lacking. Among the studies reported in the literature, some have used limited numbers of mitochondrial DNA markers [14, 16, 17], or a few selected autosomal microsatellite or single nucleotide polymorphisms (SNPs) [16, 18–20], and others have analyzed a limited number of breeds from India [21], or indicine breeds from other countries [17, 22]. Thus, relatively little is known about the genetic diversity of Indian cattle.

In this paper, we present the results of the largest sampling of Indian indigenous cattle breeds so far, which includes 15 pure breeds and a population of non-descript animals genotyped on the 777 k SNP BovineHD chip. We analyzed the within- and between-breed genetic diversity and derived past and present estimates of effective population sizes. In addition, we performed comparisons of the Indian breeds sampled here with other indicine breeds and populations outside of India and with taurine breeds.

## Methods

### Data

**Fig. 1:**
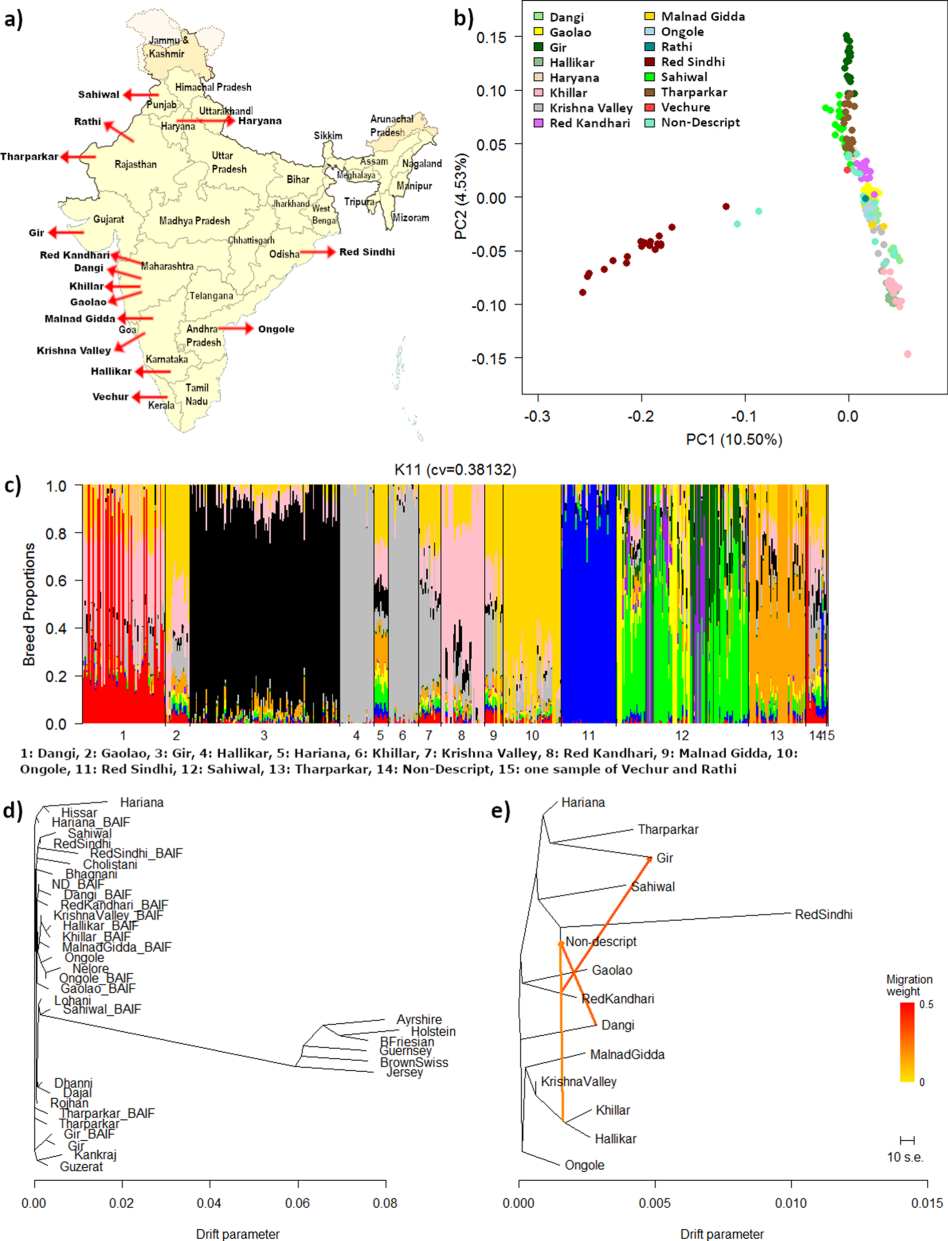
**a** Map showing the areas from which various breeds were sampled, **b** PC1 vs PC2 obtained with up to 20 animals per pure BAIF indigenous breed, **c** Estimated breed proportions of BAIF indigenous samples from an unsupervised admixture analysis with the lowest cross-validation error (CV = 0.38); K = 11. Maximum likelihood tree for **d** BAIF indigenous breeds and reference breeds (35 k data) and **e** for BAIF indigenous breeds (700 k data) with three migration edges

The BAIF Development Research Foundation sampled 699 Indian indigenous cows and bulls in 2017 from different locations across India (Fig. 1a). Samples included 68 Dangi, 20 Gaolao, 121 Gir, 28 Hallikar, 17 Hariana, 25 Khillar, 22 Krishna Valley, 35 Red Kandhari, 19 Malnad Gidda, 50 Ongole, 1 Rathi, 63 Red Sindhi, 140 Sahiwal, 48 Tharparkar, 1 Vechur, and 43 indigenous non-descript animals (ND), sampled from the Maharashtra, Odisha, Uttar Pradesh states (see Additional file 1: Table S1). Since the Red Sindhi individuals were sampled exclusively from the Central Breeding farm Chiplima in the Odisha state, and from surrounding farms that extensively use the central farm breeding stock, a high level of relationship may exist between these animals.

All animals were genotyped with the 777 k-SNP BovineHD Beadchip (Illumina Inc., San Diego) and a minimal quality control (QC) was run using the SNPQC pipeline [23] to provide high-quality genotypes and to keep as many SNPs as possible that might be able to differentiate between breeds. QC criteria included a median GC score lower than 0.6 and a call rate higher than 0.9 for each animal. Four Ongole and one Red Sindhi were excluded due to low call rates, and after QC 716,588 SNPs remained forming a set referred to as the 700 k dataset.

In addition, 345 animals from reference breeds were included in the analyses, i.e. six exotic dairy breeds including Holstein, Jersey, Canadian Ayrshire, British Friesian, Guernsey, and Brown Swiss, and 18 indicine breeds including Achai, Bhagnani, Cholistani, Dajal, Dhanni, Gabrali, Gir, Guzerat, Hariana, Hissar, Kankraj, Lohani, Nelore, Ongole, Red Sindhi, Rojhan, Sahiwal, and Tharparkar (see Additional file 1: Table S1). Only a few of these 18 indicine breeds were sampled from India, and it was uncertain whether they could represent individual reference breeds for Indian cattle. Nonetheless, we included these breeds for a comparison between publicly available data and the BAIF data collected exclusively from India and as a reference for total indicine breed proportion. The reference breeds were sourced from the HapMap consortium (700 k [24]), the Dairy Genetics East Africa Project (DGEA, 700 k [25]), from Decker et al. (50 k [26]), the Canadian Dairy Network (CDN, 700 k), and Scotland’s Rural College (SRUC) (700 k). All additional data except for the British Friesian had already been through QC. British Friesian were subjected to the same QC as the BAIF indigenous samples (above). The number of animals per reference breed was to 20 and if more than 20 animals were available from the source, we chose 20 individuals randomly.

Based on a preliminary screen of the data using a supervised analysis in Admixture [27] to obtain estimates of ancestral breed proportions of *Bos taurus* versus *Bos indicus*, we excluded indicine reference animals that had more than 1% taurine breed ancestry (see “Methods” on Admixture below), i.e. all the Achai and Gabrali individuals, nine Ongole, three Bhagnani, three Tharparkar, two Lohani, two Nelore, two Red Sindhi, and one Dajal (see Additional file 1 Table S1).

Among the 694 Indian indigenous samples that passed QC, 109 were identified as having more than 1% taurine breed ancestry, i.e. 36 Sahiwal, 27 non-descript, 20 Red Sindhi, four Hariana, five Krishna Valley, five Malnad Gidda, three Dangi, three Gir, three Tharparkar, one Gaolao, one Hallikar, and one Khillar (see Additional file 1: Table S1). These 109 animals were excluded from the final principal component analyses, the estimation of effective population size, and all the breed diversity analyses, and in the admixture analyses they were identified separately.

Merging the BAIF breeds with the reference breeds resulted in a dataset of 35,684 SNPs, hereafter referred to as the 35 k dataset. The main reduction in the number of SNPs from the 700 k dataset to the 35 k dataset is due to the 50 k data available from Decker et al. [26].

### Principal component analyses

Principal components (PC) were calculated based on the genomic relationship matrix (GRM) which was derived using two approaches. The first approach constructed the GRM according to VanRaden [28]. Missing genotypes were replaced by average genotypes across all animals. The second approach constructed the GRM according to Yang et al. [29] (see Additional file 2: Material and Methods S1). It should be noted that the GRM are constructed across multiple breeds which skews the values of diagonal and off-diagonal elements for some breeds, especially when the allele frequencies of one breed differ from those of the other breeds [25]. Therefore, we constructed four GRM using: [1] all the reference breeds plus the Indian indigenous breeds sampled in this study, [2] only the exotic reference breeds, [3] only the indicine reference breeds, and [4] only the Indian indigenous breeds. Each GRM was chosen for the appropriate follow-up analysis.

### Correlations of allele frequencies

Allele frequencies ($p$) of our sample of Indian indigenous breeds were calculated based on the 700 k dataset after excluding the animals with more than 1% *Bos taurus* ancestry, as estimated by admixture analysis. To remove potential bias due to a limited number of SNPs with a low minor allele frequency (MAF), we applied a cut-off threshold so that only SNPs with a MAF higher than 0.05 were used.

We calculated the correlation of the observed allele frequencies between each breed-pair, ${\mathrm{R}}_{\mathrm{obs}}$, according to Pearson. We combined all the Indian indigenous breeds sampled in this study and all the *Bos taurus* reference breeds together to estimate the expected allele frequencies. The correlation of the expected allele frequencies between two breeds, ${\mathrm{R}}_{\mathrm{exp}}$, which is due only to random sampling errors was estimated as:

$${{\mathrm{R}}_{\mathrm{exp}}=\mathrm{V}}_{p}/\left[{\mathrm{V}}_{p}+{\mathrm{V}}_{\mathrm{e}1}+{\mathrm{V}}_{\mathrm{e}2}\right],$$

where, ${\mathrm{V}}_{p}$ is the variance of $p$ in the meta-population (i.e. all Indian indigenous animals or all *Bos taurus* reference animals), ${\mathrm{V}}_{\mathrm{e}1}$ and ${\mathrm{V}}_{\mathrm{e}2}$ are the error variances of the estimates of $p$ in the two breeds (see Additional file 2: Material and Methods S2).

The variance of the true SNP allele frequencies in one breed that was explained by the true SNP allele frequencies in another breed was estimated as:

$${\mathrm{R}}_{\mathrm{obs}}^{2}/{\mathrm{R}}_{\mathrm{exp}}^{2},$$

and this ratio was calculated for all breed pairs within a group.

### Admixture analyses

Ancestral breed proportions of the Indian indigenous animals were estimated by using either the reduced 35 k SNP set when all reference breeds were included for a supervised admixture analysis, or the full 700 k marker set when only the 16 BAIF indigenous populations were used in an unsupervised admixture analysis [27]. The unsupervised analysis was run for K (number of ancestral populations) = 2 to 16 and the lowest cross-validation error was obtained for K = 11 (CV error = 0.381). The f3 statistic [30, 31], as implemented in the TreeMix software [32], was used to analyze additional admixture in the Indian indigenous populations based on 35 blocks of 1000 SNPs.

### Breed diversity and phylogeny

Breed diversity was measured based on allele frequencies, the GRM (described above according to VanRaden [28]), and ${F}_{\mathrm{IS}}$ and ${F}_{\mathrm{ST}}$ values that were calculated according to Nei [33] and Weir and Cockerham [34], respectively. Since GRM elements tend to be biased when they are calculated across multiple distinct breeds (such as taurine and indicine breeds, [25]), we set up separate GRM for the exotic taurine breeds, the reference indicine breeds, and the BAIF indigenous breeds, as described above.

Runs of homozygosity (ROH) were also analyzed in the Indian indigenous animals to assess inbreeding, using the R-package detectRUNS with a sliding-window approach similar to that of Purcell and Neale [35]. According to the recommendations by Meyermans et al. [36], we did not prune the data for linkage disequilibrium (LD) or MAF. We used a window size of 20 SNPs and a window-threshold of 0.05, a minimum ROH length of 20 SNPs within at least 25 kb, and a maximum gap size between SNPs of 75 kb.

Phylogenetic trees were constructed with the TreeMix v.1.72.0 software [32]. Maximum likelihood trees were constructed using all the breeds including all the reference breeds, or including only the Indian indigenous breeds sampled in this study. With the Indian indigenous breeds, 0 to 14 migration events were tested with 10 iterations per migration event. The best number of migration events was determined with the R package OptM [37]. The best fit to the data based on the Akaike information criterion (AIC) was provided by a simple exponential model followed by a non-linear least squares model. Both models predicted three migration events as the most likely number. Blocks of 1000 SNPs were used in both analyses to account for dependencies between nearby markers.

The f4 statistic [30, 31], as implemented in TreeMix [32], was used to test whether two clusters of four populations have a significant gene flow between the clusters (A,B;C,D). Blocks of 1000 SNPs were also used for the f4 statistic.

### Effective population sizes

We estimated the effective population size (${N}_{e}$) for the Indian indigenous breeds that had to fulfill two additional criteria to ensure the most accurate estimates as possible: (1) for each pair of animals, if they had an off-diagonal GRM value higher than 0.2, one member of the pair was excluded from the LD calculations to ensure that each breed was represented by a reasonably unrelated sample; and (2) each breed sample had to have more than 20 individuals to reduce bias. Thus, finally, only the Dangi, Gir, Hallikar, Khillar, Ongole, and Sahiwal breeds remained for the estimation of ${N}_{e}$. However, we also calculated the effective population size of the other Indian indigenous breeds (see Additional file 11: Figure S6), but these ${N}_{e}$ estimates should be interpreted with caution, since the number of individuals per breed was small (< 20). The 700 k SNP data was pruned to include only SNPs with a MAF higher than 0.05, which resulted in 380,856 remaining SNPs that were used to calculate LD between makers separated by 0 to 1 Mb. A second dataset with 54 k SNPs, in which every 7^th^ marker was retained, was used to calculate LD between markers separated by 1 to 50 Mb in order to speed up computing time without losing too much accuracy. The LD calculations are described in Material and Methods S3 (see Additional file 2: Material and Methods S3).

To determine the decay of LD with increasing distance between SNPs, the average ${r}^{2}$ within each breed was expressed as a function of the distance between pairs of SNPs. Pairs of SNPs were grouped by their pairwise distance into intervals of 10 kb, starting from 0 up to 10 Mb, and the average ${r}^{2}$ for all pairs of SNPs in each interval was estimated.

The ${r}^{2}$ values combined with distances between markers were used to estimate the effective population size (${N}_{e}$) at a given point in the past, assuming a model without mutation and using the formula described by Sved [38] and extended by Weir and Hill [39]:

$${N}_{e}=\left(\frac{1}{4c}\right)\left(\frac{1}{{r}_{adj}^{2}}-1\right),$$

where ${N}_{e}$ is the effective population size, $c$ the distance between markers in Morgan (assuming 1 cM = 1 Mb), and ${r}_{adj}^{2}={r}^{2}-(1/2*\mathrm{n})$. The time (number of generations) at which ${N}_{e}$ was estimated is given as $1/2c$ [40]. For distances between markers ranging from 0 to 2 Mb, the data were grouped into 80 bins of 25 kb to calculate ${N}_{e}$ in generations 26 to 2000; for distances between markers ranging from 2 to 50 Mb, data were grouped into 25 bins of $1/2*$ 100 Mb to calculate ${N}_{e}$ in generations 1 to 25. The binning process was designed to ensure a sufficient number of pairs of SNPs within each bin and to obtain a representative average ${r}_{adj}^{2}$.

## Results and discussion

### Principal component analyses

The two GRM that were tested produced almost identical results with differences of a magnitude lower than 0.001 in their diagonal or off-diagonal elements per breed. Accordingly, principal components differed by only 1.31% for the first PC (PC1) and by less than 0.34% for PC2. Many livestock-related studies use GRM for genome-wide association studies (GWAS) or genomic prediction purposes based on VanRaden’s method rather than only for genetic diversity studies. Thus, our study focuses on the results from the first approach based on VanRaden’s method [28], to make it more applicable for future potential genomic improvement studies in Indian cattle.

**Fig. 2:**
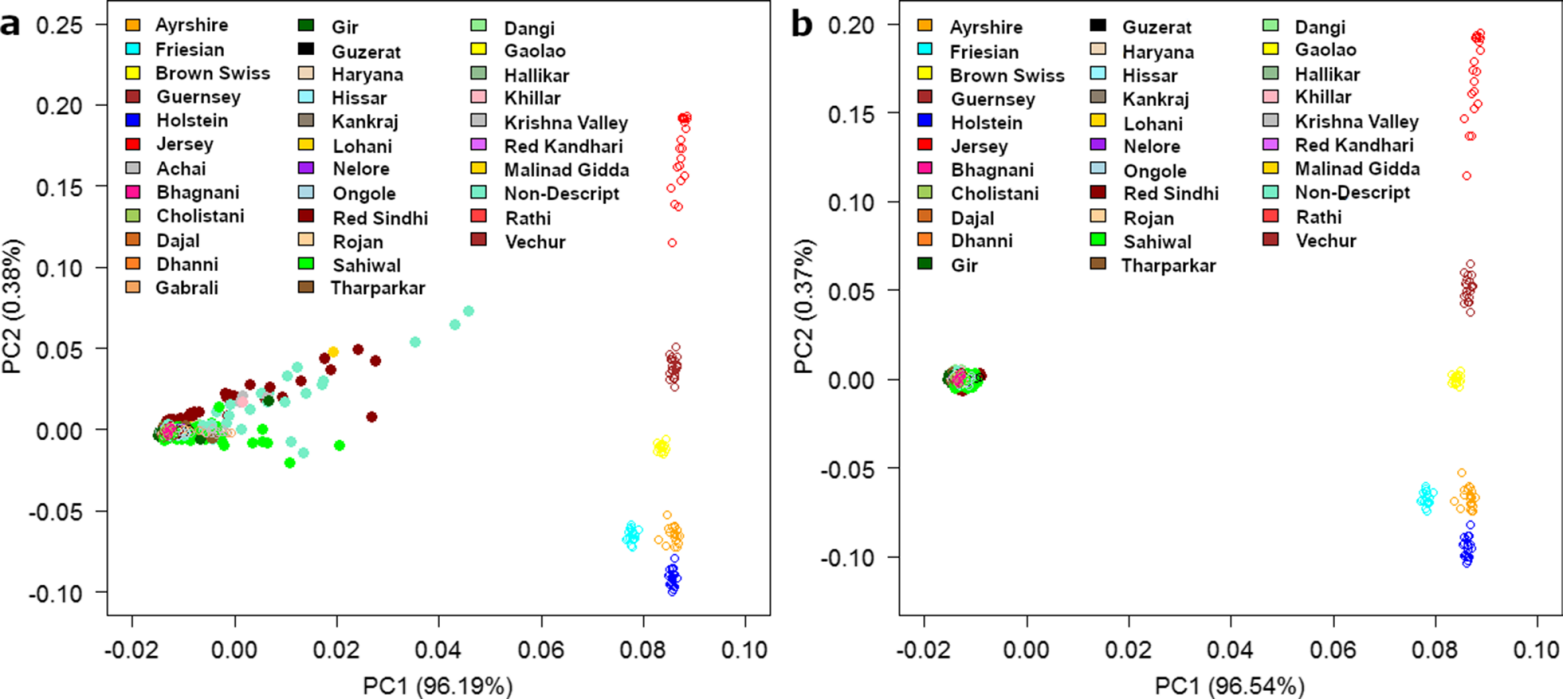
PC1 and PC2 with 35 k SNPs **a** with reference (open circle) and all BAIF indigenous breeds (filled circles) and **b** when BAIF indigenous animals with more than 1% taurine ancestry were removed

PC1 differentiated *Bos indicus* from *Bos taurus* breeds. Several of the Indian indigenous breeds in our study, especially Sahiwal, Red Sindhi, and the non-descript population, were distributed towards the taurine reference breeds (Fig. 2a). A similar result was presented by Nayee et al. [21] who also found some Red Sindhi animals that break away from the tight indicine cluster towards a Jersey reference breed. This shows that a proportion of animals that, phenotypically, appear to be pure indicine can have substantial proportions of *Bos taurus* ancestry. The same result was found when sampling breeds in East Africa where breed purity could not be determined based on phenotypic features, i.e. some of the animals that had a crossbred phenotype were shown to be nearly pure indigenous and some of the animals sampled as pure indigenous were shown to be crossbred after genotyping [25, 41].

After excluding indicine animals (reference animals and our sample) that had more than 1% taurine content from the PC analysis, all the Indian indigenous and indicine reference breeds clustered tightly together **(**Fig. 2b). In the PC analysis, the classes with the largest number of animals tended to dominate the results, which in this case are the *Bos indicus* samples. In spite of this expected bias, a single tight cluster was found for all the *Bos indicus* breeds compared to the six *Bos taurus* breeds, confirming the results reported by Gajjar et al. [42]. To create the 700 k SNP and especially the 50 k SNP chips, SNPs with a high MAF across several *Bos taurus* breeds were favored, which results in a bias that should reduce the observed variation in SNPs between *Bos taurus* breeds compared to *Bos indicus* breeds. Thus, the clustering of breeds indicates that substantially more molecular genetic variation exists between *Bos taurus* than between *Bos indicus* breeds.

Using only the Indian indigenous animals (700 k data) sampled for this study, the first six PC were obtained with up to 20 animals per breed and by removing all admixed animals (Fig. 1b) and (see Additional file 3: Figure S1). The first PC separated the Red Sindhi and two non-descript animals from all the other animals and showed a substantial amount of variation within the Red Sindhi breed. Similarly, PC2 through PC6 separated breeds and animals within breeds simultaneously, which is in marked contrast to the earlier PC analyses of *Bos taurus* breeds (e.g. see Fig. 2) that revealed tight clusters of animals within breeds. The creation of the BovineHD 777 k-SNP Genotyping Illumina BeadChip resulted in less bias towards *Bos taurus* SNPs than most of the lower density assays, because it is based on data from three *Bos indicus* breeds (104 animals) and 20 *Bos taurus* breeds (452 animals). However, selection of markers was heavily biased towards SNPs that have a high MAF within and across breeds and put much more emphasis on *Bos taurus* data, such that a bias against diversity of *Bos taurus* rather than between *Bos indicus* breeds is expected. Thus, these results also point strongly to much less molecular genetic variation between *Bos indicus* breeds than between *Bos taurus* breeds.

The existence of occasional outliers, such as a single Malnad Gidda animal on PC6 and a single Khillar animal on PC8, 9 and 10, suggests that there may be additional breed variation that is not captured by the current sampling of these breeds. Indeed, these two outliers could possibly represent animals that belong to other (unknown) breeds and were misclassified in the field as Malnad Gidda and Khillar.

### Correlations of SNP allele frequencies between breeds

For all the breeds genotyped on both the 35 k and 700 k SNP sets, the average allele frequency was lower than 0.5, with the largest bias being observed for the 35 k SNP set and for the indicine breeds (see Additional file 4: Figures S2 and Additional file 5: Figure S3), as previously reported [25, 43], but its cause is unknown.

**Fig. 3:**
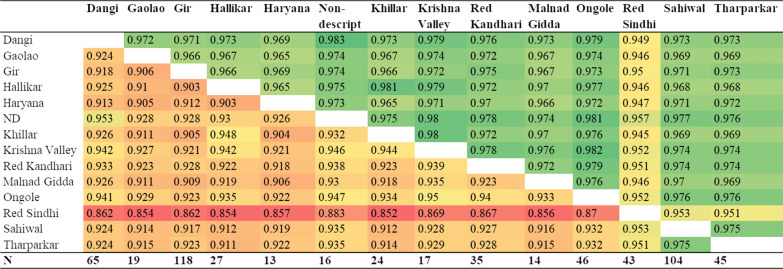
Correlations of SNP allele frequencies between indigenous breeds based on the 700 k data (color scale from red to green for the smallest to largest values). *Above the diagonal: using all SNPs and below the diagonal using SNPs with MAF > 0.05

**Fig. 4:**
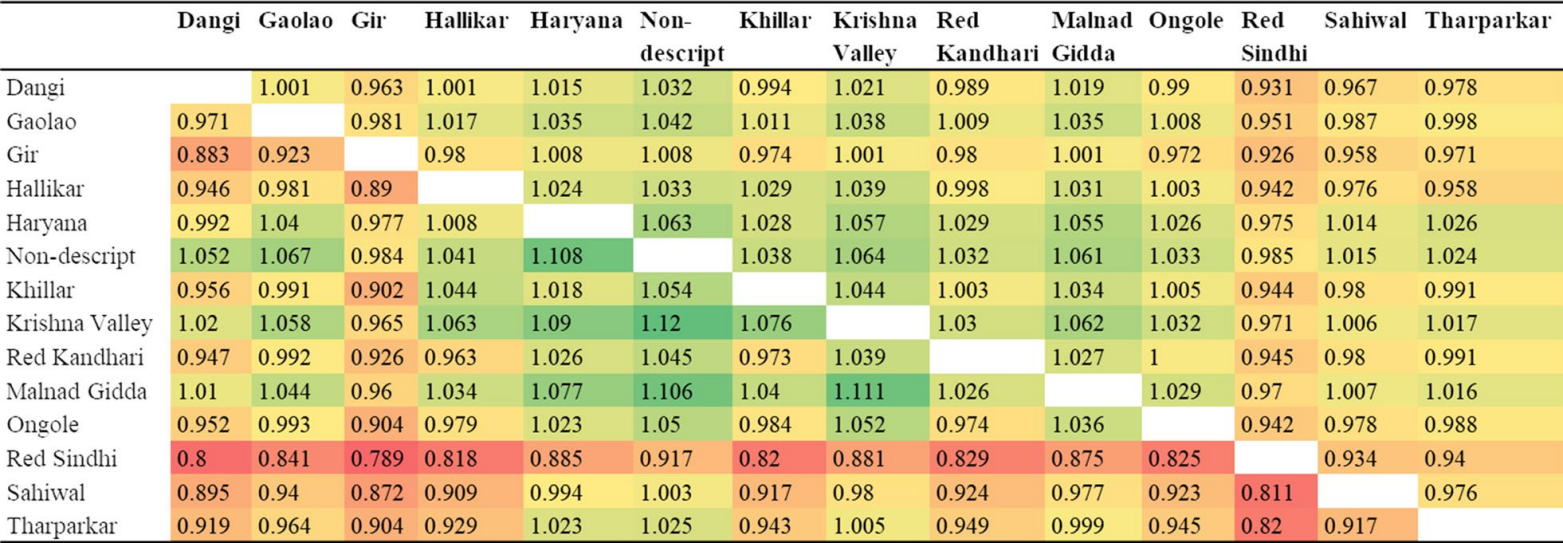
${\mathrm{R}}_{\mathrm{obs}}^{2}/{\mathrm{R}}_{\mathrm{exp}}^{2}$ as an estimate of the proportion of variance in true allele frequency in one breed explained by the true allele frequencies in another breed based on the 700 k data (color scale from red to green for the smallest to largest values). *Above the diagonal: using all SNPs and below the diagonal: using SNPs with a MAF > 0.05. Estimates less than 1.0 are shaded

The correlation of SNP allele frequencies between breeds provides an assessment of whether a purpose-designed assay for Indian indigenous breeds should take differences in SNP allele frequencies between breeds into account. Figure 3 shows the correlations of allele frequencies between the Indian indigenous breeds in our dataset, based on the 700 k assay, with all SNPs included above the diagonal of the table and when only using SNPs with a MAF higher than 0.05. The last row of Fig. 3 shows the number of animals included in each breed sample. The correlations of allele frequencies obtained by excluding SNPs with a MAF lower than 0.05 are also included to minimize the bias that could be caused by the large number of SNPs on the 700 k assay that are known to have extreme allele frequencies in *Bos indicus* breeds. Even if the true allele frequencies are the same in all the populations, the observed correlations in Fig. 3 are expected to be lower than 1.0 due to random sampling from finite populations. The estimated proportion of the variance in true allele frequencies in one breed that is explained by the true variance in another breed, ${\mathrm{R}}_{\mathrm{obs}}^{2}/{\mathrm{R}}_{\mathrm{exp}}^{2}$, is in Fig. 4, for the same data combinations as in Fig. 3. The average proportion of variance within breeds is 1.002 when all SNPs are included and 0.986 when only SNPs with a MAF higher than 0.05 are included, which shows that most of the variance in allele frequencies is within breeds and very little between breeds.

The correlations of each breed’s allele frequencies with the average allele frequency across the whole sample of 586 Indian indigenous animals ranged from 0.967 to 0.990 for all SNPs. Because the ascertainment bias of the SNP assay resulted in more markers with a low MAF in the *Bos indicus* breeds than in the *Bos taurus* breeds, we also calculated the correlations by excluding SNPs with a MAF lower than 0.05 and found that it ranged from 0.910 to 0.972. The lowest correlation, i.e. 0.91, was obtained for Red Sindhi, whereas the correlations for all the other breeds were higher than 0.95. While these correlations involve part-whole relationships (the allele frequency of each breed contributes to the average allele frequency across all breeds), the high correlation values indicate that individual breeds are well represented in the average frequency across all indigenous animals.

For a comparison with the correlations of each breed’s allele frequencies with the average allele frequency across all indigenous animals, Additional file 6: Table S2 shows the correlations of allele frequencies for the *Bos taurus* reference breeds, based on 700 k data. This table shows that whereas expected correlations of 0.902 and 0.864, respectively above and below the diagonal, were obtained based on a sample size of 20 randomly chosen animals for each breed, all the observed correlations were much lower, which indicates that substantial differences exist between breeds. The ${\mathrm{R}}_{\mathrm{obs}}^{2}/{\mathrm{R}}_{\mathrm{exp}}^{2}$ ratio ranges from 0.539 to 0.783 (average 0.644) when all SNPs are included, and from 0.462 to 0.742 (average 0.576) when SNPs with a MAF higher than 0.05 are included. This indicates that for the *Bos taurus* dairy breeds, about 60% of the variance in SNP allele frequency is within breeds and about 40% between breeds.

Thus, in conclusion the Indian indigenous *Bos indicus* breeds can be treated as belonging to a single population for the purpose of designing an SNP assay, while the *Bos taurus* breeds that show substantial differences in SNP allele frequency between breeds should be taken into account separately for an optimal assay design.

### Admixture analyses

For assigning ancestral proportions, unsupervised admixture analyses use the information directly from the target population. Thus, the composition of the target population affects the admixture estimates, which makes a direct comparison of ancestral proportions between studies difficult. However, unsupervised admixture analyses are not intended to indicate true ancestral breed composition but are useful to indicate the diversity between and within breeds and the degree of affinity between the various *Bos indicus* reference samples and the Indian indigenous animals in our dataset. However, given the very large genetic distance between the *Bos taurus* and *Bos indicus* breeds, the admixture analyses do provide an accurate estimate of the proportion of *Bos taurus* versus *Bos indicus* admixture.

**Fig. 5:**
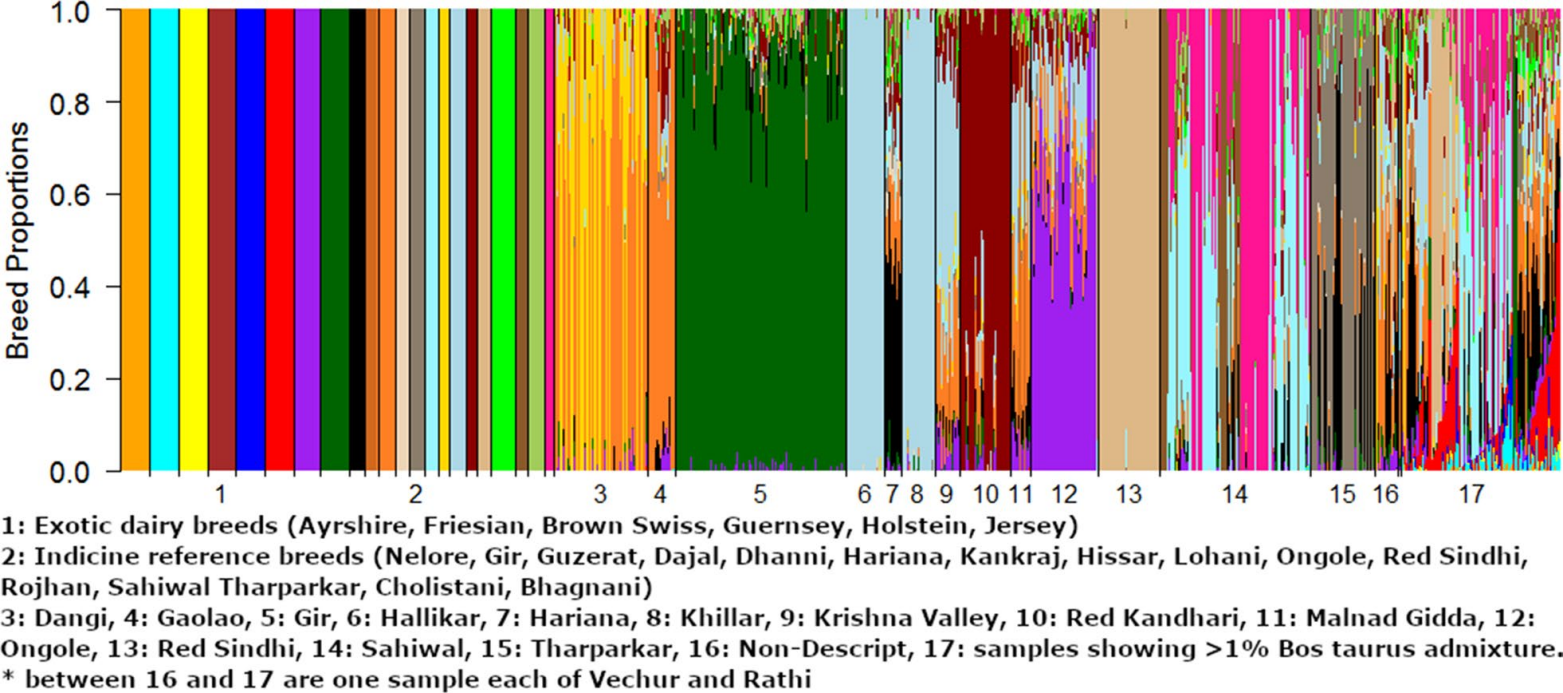
Estimated breed proportions of the BAIF indigenous samples from a supervised admixture analysis using exotic dairy breeds and *Bos indicus* reference breeds. 1: Exotic dairy breeds (Ayrshire, Friesian, Brown Swiss, Guernsey, Holstein, Jersey). 2: Indicine reference breeds (Nelore, Gir, Guzerat, Dajal, Dhanni, Hariana, Kankraj, Hissar, Lohani, Ongole, Red Sindhi, Rojhan, Sahiwal Tharparkar, Cholistani, Bhagnani). 3: Dangi, 4: Gaolao, 5: Gir, 6: Hallikar, 7: Hariana, 8: Khillar, 9: Krishna Valley, 10: Lal Kandhari, 11: Malnad Gidda, 12: Ongole, 13: Red Sindhi, 14: Sahiwal, 15: Tharparkar, 16: non-descript population, 17: samples showing more than 1% *Bos taurus* admixture. *between the non-descript (16) and the admixed population (17) are one sample each of the Vechur and Rathi breeds

Figure 5 shows the estimated ancestral breed proportions of the Indian indigenous animals from a supervised admixture analysis in which all six European *Bos taurus* and 16 *Bos indicus* reference breeds are set as a possible ancestral breed. This analysis found that 109 of the Indian indigenous samples contained more than 1% *Bos taurus* admixture (average 12.9%; SD 12.5%; range 1.0 to 60.2%). The proportion of animals with more than 1% *Bos taurus* admixture ranged from 0 (Ongole and Red Kandhari) to 0.63 (non-descript individuals) and the proportions for the other breeds were the following: Red Sindhi (0.32), Sahiwal (0.26), Malnad Gidda (0.26), Haryana (0.24), Krishna Valley (0.23), Tharparkar (0.06), Gaolao (0.05), Dangi (0.04), Khillar (0.04), Hallikar (0.04), and Gir (0.02). Among the remaining 588 Indian indigenous animals with less than 1% *Bos taurus* admixture, 578 were estimated to have less than 0.01% *Bos taurus* admixture. This strong dichotomy between animals with and without *Bos taurus* admixture and the large variation in admixture proportion for animals with more than 1% *Bos taurus* admixture is consistent with admixture being of very recent origin. An ancient admixture in the history of the development of breeds post-domestication would have led to a near equal *Bos taurus* proportion in all the animals of breeds that had an ancient admixture.

**Fig. 6:**
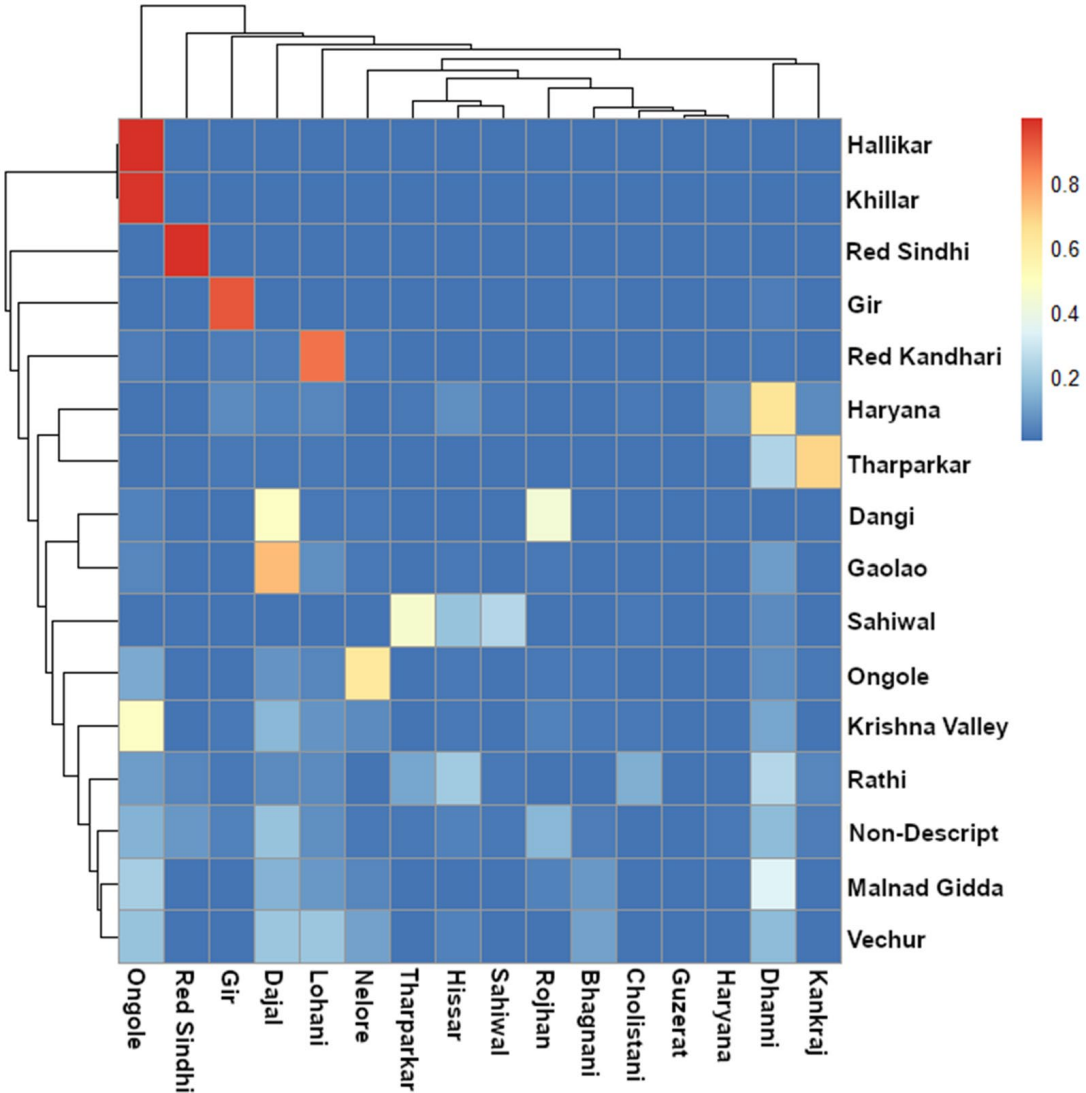
Heatmap of the estimated breed proportion estimates of the BAIF indigenous samples (rows) from a supervised admixture analysis using only *Bos indicus* reference breeds (columns)

Figure 6 shows the estimated ancestral breed proportions of all Indian indigenous animals (excluding animals with a *Bos taurus* admixture > 1%) from a supervised admixture analysis when only the indicine reference breeds are set as possible ancestral breeds. The proportion of each ancestral reference breed (columns) is shown for each Indian indigenous breed sampled in our study. A number of Indian indigenous breeds appear to have a high affinity with individual indicine reference breeds. For example, the origin of the indigenous Gir and Red Sindhi samples appears to be primarily of the reference Gir and Red Sindhi breeds, respectively. The origin of the Khillar and Hallikar animals appears to be mostly of the reference Ongole breed and that of the indigenous Tharparkar sample appears to be mostly of the reference Kankraj breed, with a small contribution from the reference Tharparkar breed. In addition, a number of the Indian indigenous breeds in our sample include small contributions from many reference breeds. Consistent with its name, the origin of the non-descript population showed little affinity with any of the reference breeds.

The heatmap patterns in Fig. 6 show some interesting within-breed variations. For example, 23 of the 45 Indian indigenous Tharparkar animals have an estimated 100% Kankraj ancestry whereas most of the 22 remaining animals have less than 30% Kankraj ancestry of which five animals show more than 80% estimated Dhanni ancestry and most of the 17 others show 20 to 50% Dhanni ancestry. Among the 65 Dangi animals, 22 and 34 were estimated to have more than 99% Rojhan ancestry and more than 60% Dajal ancestry, respectively. This incomplete alignment between reference breeds and the indigenous breed sampled here may reflect an inaccurate assignment of the reference breeds, most of which were sampled outside India, and/or a poor ability of assigning breeds, which is more generally due to the relatively small amount of molecular genetic variation between breeds (see later).

Figure 1c shows the estimates of ancestral contributions obtained with the unsupervised admixture model and the lowest cross-validation error (CVE = 0.38 for K = 11). The plots for the unsupervised analysis with K ranging from 2 to 11 and their CVE are in Additional file 7: Figure S4 and Additional files 8: Figure S5. The CVE value decreases only a little at K greater than 6, thus interpretation of more than six ancestors should be made with caution. These unsupervised analyses support the patterns obtained in the supervised analyses. For example, the Gir animals show a very distinctive signal that is hardly shared with the other breeds but clearly shared with the reference sample of Gir (Figs. 3 and 4). Nayee et al. [21] found that their Gir sample was split into a pure population and a population with about one third of admixture made up of the other Indian indigenous breeds used in their study. Similarly, Red Sindhi also showed a very similar pattern of distinct breed composition, in agreement with the results of Nayee et al. [21]. The Hallikar and Khillar breeds share a common signal (Fig. 1c) that matches that of the indicine reference for Ongole in the supervised analyses (Figs. 5 and 6). This single distinct signal for Hallikar and Khillar stands in contrast to the findings of Gajjar et al. [42] who reported admixture of these two breeds related to Amrithmahl, Ongole, Deoni, and Kankraj. For the Dangi breed, two distinct groups of animals are observed, one with a strong red signal (Fig. 1c), which corresponds to Rojhan in the supervised analyses (Figs. 5 and 6), and the other with a more mixed signal (Fig. 1c) that has more than 50% Dajal ancestry in the supervised analyses (Figs. 5 and 6). The Sahiwal animals show a highly heterogeneous pattern with four main groups that have high proportions of either the yellow, light green, purple or dark green signals, none of which appear as large proportions in any other breed (Fig. 3). This result for the Sahiwal breed is in contrast to those of Nayee et al. [21] and Gajjar et al. [42] who reported Sahiwal as of the breeds with the least evidence of admixture. However, it is not clear whether sampling locations differed between studies, which would be the most plausible explanation for the discrepancies between the results. Our supervised analyses, which included exotic dairy reference breeds, showed that the three main ancestral breeds of the Sahiwal breed were Bhagnani, Hissar and Tharparkar (Fig. 5), but the analysis that included only the indicine reference breeds showed that the three ancestral breeds were Sahiwal, Tharparkar and Hissar (Fig. 6). Our results for the other breeds appear to agree with those of Gajjar et al. [42] regarding a manifold admixture of Hariana and the relatively distinct signal for Ongole and Tharparkar.

The f3 test determines whether a population A shows admixture based on populations B and C (A;B,C). Significant Z-scores (< −3) were found only for the non-descript population, which shows admixture from ten of the other Indian indigenous breeds (excluding Gaolao, Red Kandhari, and Malnad Gidda), which agrees with the results of the admixture analysis (see Additional file 9).

### Breed diversity measures

Table 1 shows the estimates of ${F}_{\mathrm{IS}}$ for the reference and indigenous breeds based on the reduced 35 k data and the 700 k data, which gave almost identical estimates. ${F}_{\mathrm{IS}}$ is a measure of the relative degree of inbreeding of individuals within a breed, with higher values indicating a higher degree of inbreeding. Our estimates of ${F}_{\mathrm{IS}}$ indicate that inbreeding levels are low in all the breeds studied here. It should be noted that, because the estimates in Table 2 are derived within the *Bos taurus* or within the *Bos indicus* datasets considered as separate datasets, the ascertainment bias towards high MAF for *Bos taurus* SNPs on the 35 k and 700 k assays does not bias these parameter estimates. Our results are in contrast to those published by Sharma et al. [16] or Mukesh et al. [44] who used microsatellite markers to estimate ${F}_{\mathrm{IS}}$ and reported significant inbreeding levels in several Indian indigenous breeds. While the absolute ${F}_{\mathrm{IS}}$ values cannot be directly compared between studies that use different datasets, our results agree with those of Sharma et al. [16] regarding the highest ${F}_{\mathrm{IS}}$ values for the Gaolao and Ongole breeds and comparatively low values for the Hariana breed, whereas Mukesh et al. [44] found high ${F}_{\mathrm{IS}}$ values for the latter breed. This demonstrates that estimates of genetic diversity are relatively variable for Indian indigenous cattle.

**Table 1:** ${F}_{\mathrm{IS}}$(± SD) based on the 35 k and 700 k data, and average diagonal and off-diagonal elements of the GRM

Group	Breeds	*F*_*IS*_ 35 k	*F*_*IS*_ 700 k	GRM diagonal	GRM off-diagonal
Exotic dairy reference	Ayrshire	− 0.029 (± 0.203)	− 0.026 (± 0.205)	1.087 (± 0.048)	0.226 (± 0.076)
	Friesian	− 0.005 (± 0.21)	− 0.006 (± 0.212)	1.068 (± 0.023)	0.128 (± 0.051)
	Brown Swiss	− 0.02 (± 0.2)	− 0.02 (± 0.2)	1.096 (± 0.033)	0.269 (± 0.073)
	Guernsey	0.02 (± 0.217)	0.023 (± 0.218)	1.112 (± 0.04)	0.246 (± 0.098)
	Holstein	− 0.026 (± 0.205)	− 0.022 (± 0.209)	1.139 (± 0.039)	0.22 (± 0.093)
	Jersey	− 0.004 (± 0.213)	− 0.001 (± 0.217)	1.115 (± 0.038)	0.313 (± 0.116)
Indicine reference	Bhagnani	0.027 (± 0.319)	0.027 (± 0.319)	1.096 (± 0.08)	0.138 (± 0.117)
	Cholistani	− 0.006 (± 0.261)	− 0.006 (± 0.261)	1.07 (± 0.116)	0.2 (± 0.159)
	Dajal	− 0.008 (± 0.299)	− 0.008 (± 0.299)	1.033 (± 0.014)	0.07 (± 0.175)
	Dhanni	0.007 (± 0.25)	0.007 (± 0.25)	1.019 (± 0.024)	0.048 (± 0.08)
	Gir	− 0.002 (± 0.21)	− 0.002 (± 0.215)	1.042 (± 0.039)	0.111 (± 0.119)
	Guzerat	0.01 (± 0.268)	0.014 (± 0.275)	1.043 (± 0.057)	0.084 (± 0.086)
	Hariana	0.001 (± 0.346)	0.001 (± 0.346)	1.189 (± 0.117)	0.459 (± 0.32)
	Hissar	0.026 (± 0.308)	0.026 (± 0.308)	1.054 (± 0.08)	0.065 (± 0.175)
	Kankraj	− 0.047 (± 0.256)	− 0.047 (± 0.256)	1.026 (± 0.02)	0.168 (± 0.139)
	Lohani	0.04 (± 0.305)	0.04 (± 0.305)	1.06 (± 0.081)	0.042 (± 0.097)
	Nelore	0.004 (± 0.208)	0.006 (± 0.215)	1.055 (± 0.039)	0.146 (± 0.068)
	Ongole	0.012 (± 0.271)	0.012 (± 0.271)	1.041 (± 0.022)	0.079 (± 0.127)
	Red Sindhi	0.052 (± 0.306)	0.052 (± 0.306)	1.044 (± 0.086)	0.043 (± 0.083)
	Rojhan	0.036 (± 0.289)	0.036 (± 0.289)	1.046 (± 0.071)	0.029 (± 0.078)
	Sahiwal	0.012 (± 0.218)	0.012 (± 0.218)	1.061 (± 0.058)	0.111 (± 0.06)
	Tharparkar	0.006 (± 0.283)	0.006 (± 0.283)	1.021 (± 0.045)	0.081 (± 0.08)
BAIF indigenous	DANGI	− 0.013 (± 0.119)	− 0.015 (± 0.124)	1.004 (± 0.021)	0.07 (± 0.091)
	GAOLAO	0.022 (± 0.22)	0.022 (± 0.226)	1.05 (± 0.069)	0.082 (± 0.115)
	GIR	0.013 (± 0.106)	0.012 (± 0.101)	1.012 (± 0.041)	0.074 (± 0.053)
	HALLIKAR	0.001 (± 0.175)	0.002 (± 0.179)	1.023 (± 0.015)	0.087 (± 0.028)
	HARIANA	− 0.0001 (± 0.249)	0.0002 (± 0.262)	1.007 (± 0.02)	0.047 (± 0.058)
	Non-Descript	0.028 (± 0.23)	0.029 (± 0.236)	1.012 (± 0.058)	0.011 (± 0.052)
	KHILLAR	0.015 (± 0.195)	0.015 (± 0.2)	1.033 (± 0.043)	0.082 (± 0.068)
	KRISHNA VALLEY	0.007 (± 0.213)	0.004 (± 0.218)	0.999 (± 0.023)	0.026 (± 0.042)
	RED KANDHARI	0.009 (± 0.165)	0.008 (± 0.168)	1.016 (± 0.05)	0.062 (± 0.087)
	MALNAD GIDDA	− 0.001 (± 0.264)	0.001 (± 0.274)	1.014 (± 0.026)	0.058 (± 0.185)
	ONGOLE	0.027 (± 0.154)	0.028 (± 0.153)	1.026 (± 0.07)	0.053 (± 0.047)
	RED SINDHI	− 0.032 (± 0.153)	− 0.034 (± 0.154)	1.072 (± 0.049)	0.225 (± 0.113)
	SAHIWAL	0.013 (± 0.109)	0.015 (± 0.108)	1.035 (± 0.067)	0.067 (± 0.086)
	THARPARKAR	− 0.022 (± 0.142)	− 0.024 (± 0.144)	0.993 (± 0.021)	0.075 (± 0.088)

Standard deviations of the ${F}_{\mathrm{IS}}$ estimates based on the 35 k and 700 k data are equal to 0.0006–0.002 and 0.0001–0.0004, respectively

GRM for the exotic breeds, indicine reference breeds, and BAIF indigenous breeds are built separately

**Table 2:** Estimates of ${N}_{e}$ at 2000, 5, and 1 generations before present

	n	Generation 2000	Generation 5	Generation 1
Dangi	27	3181	199	43
Gir	51	3077	467	197
Hallikar	26	3102	1046	399
Khillar	21	3151	394	133
Ongole	36	3242	604	151
Sahiwal	35	3282	231	68
Gaolao^a^	12	2025	64	13
Haryana^a^	8	1298	7	1
Krishna Valley^a^	15	2292	150	30
Red Kandhari^a^	15	2235	157	35
Malnad Gidda^a^	11	2136	71	14
Red Sindhi^a^	6	1400	25	6
Tharparkar^a^	15	2172	99	21
Non-descript^a^	13	2217	82	17

^a^${N}_{e}$ probably underestimated due to the small sample size

Table 1 also shows the averages of the diagonal and off-diagonal elements of the GRM for each breed. Because the GRM involves a normalization process to adjust for the distribution of allele frequencies, a GRM built across very different populations can exhibit extreme values; thus, the GRM that we constructed here considered the exotic reference breeds (700 k data except for Brown Swiss), the indicine reference breeds (mostly 35 k data) and our samples of Indian indigenous breeds (all 700 k data), separately. For the exotic breeds, we found modest levels of inbreeding (diagonal elements greater than 1) and relatively high average relationships within breeds (off-diagonal elements), as expected when there is a high degree of genetic variation between breeds and close relationships among the animals within a breed. The Indian indigenous samples generally showed relatively low inbreeding levels (diagonal elements close to 1) and quite low average relationship values between animals within a breed, which indicates a lower level of genetic differentiation between breeds and relatively more distant relationships among animals within breeds. A notable exception is the Red Sindhi breed, which has higher average diagonal and much higher off-diagonal elements than any other of the indigenous breeds. This likely reflects that the Red Sindhi animals were sampled from a single central breeding farm and its surrounding farms, where the animals are likely to be closely related to each other. The indicine reference samples generally had higher diagonal and off-diagonal elements than the Indian indigenous samples. This likely reflects the generally small size of the samples and the likelihood that these small numbers of samples were collected from a single or a small number of locations, which would generate groups of animals that are more closely related than the average for those reference breeds.

**Fig. 7:**
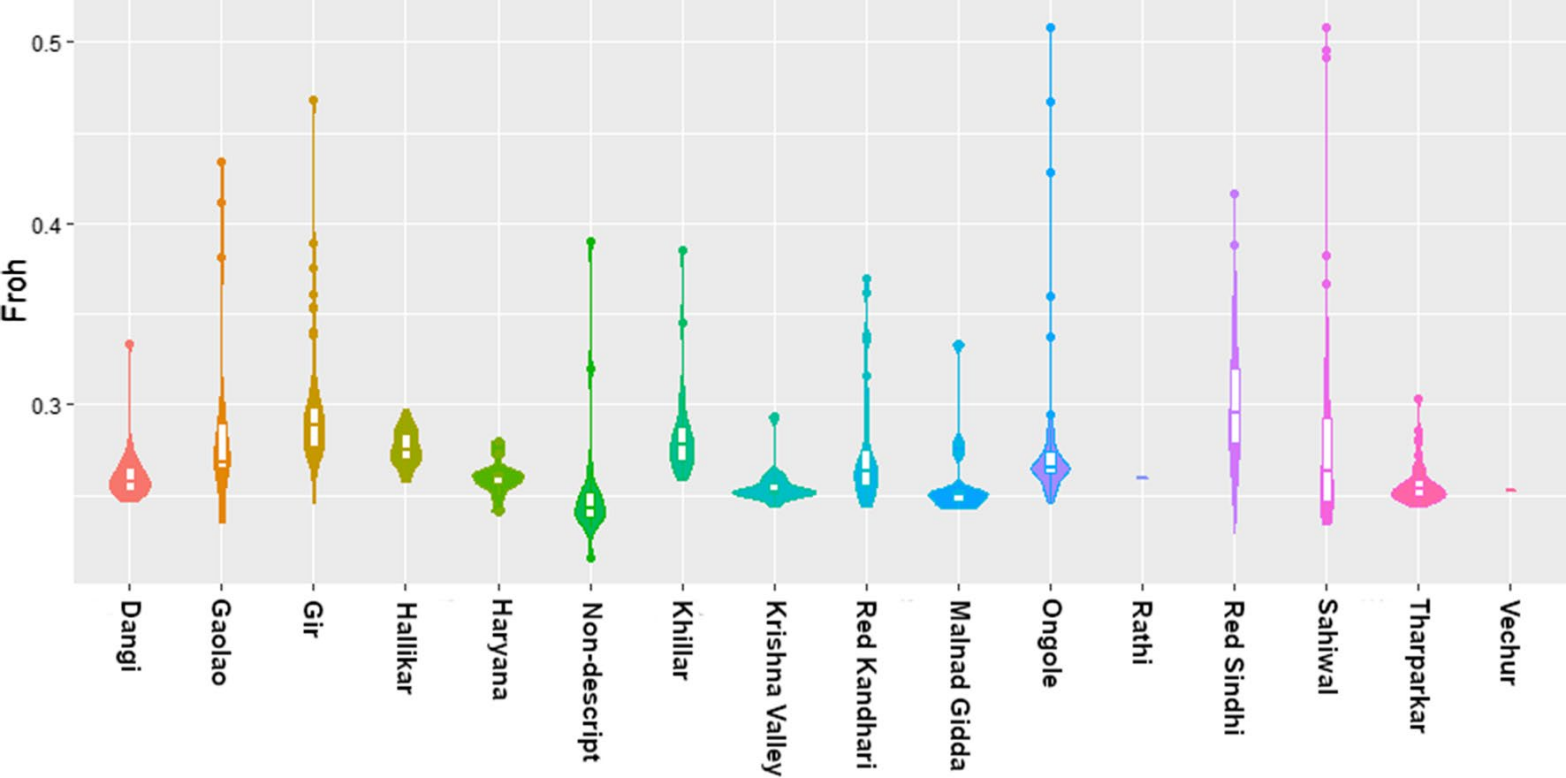
Violin plot of the inbreeding coefficients based on runs of homozygosity (${F}_{\mathrm{ROH}}$) in BAIF indigenous cattle populations

Between 99.2 and 99.9% of the detected runs of homozygosity (ROH) were smaller than 6 Mb in all of the Indian indigenous breeds sampled here. This abundance of short ROH confirms that recent inbreeding levels are low. However, the inbreeding coefficient derived from ROH (${F}_{\mathrm{ROH}}$) is on average 0.270 (± 0.016) across all Indian indigenous populations, with the smallest values found for the non-descript population (0.257) and largest values for Red Sindhi (0.302, Fig. 7). Because ${F}_{\mathrm{ROH}}$ can be interpreted as a probability of being identical-by-descent [45], the combination of low ${F}_{\mathrm{IS}}$ and large ${F}_{\mathrm{ROH}}$ values points to relatively small effective population sizes for the BAIF indigenous breeds. Most breeds include several outliers with higher ${F}_{\mathrm{ROH}}$ values, which indicates that some individuals are substantially inbred. Inbred individuals should be identified and excluded from further breeding to ensure the survival of the breed.

**Fig. 8:**
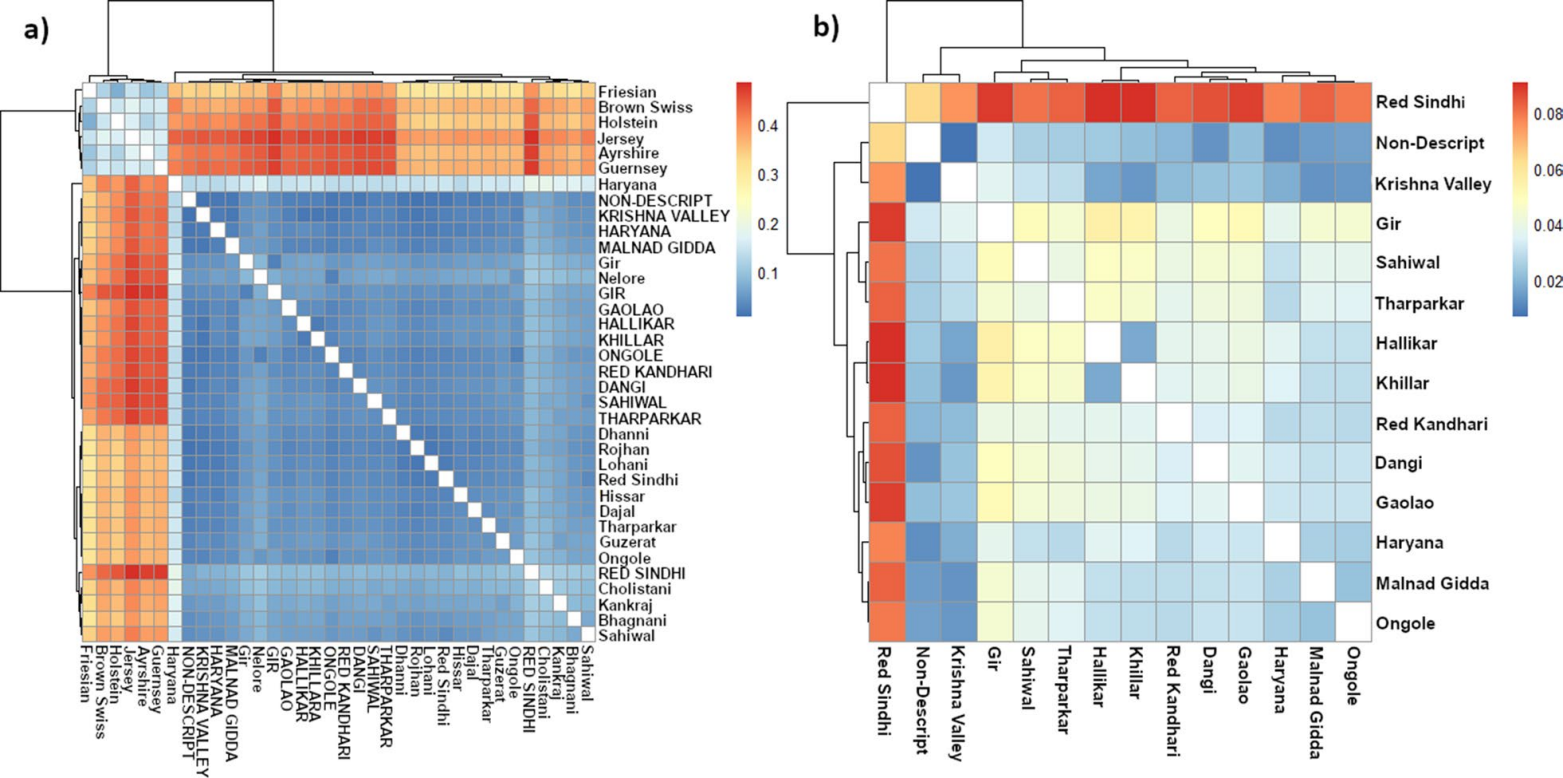
Heatmap of ${F}_{\mathrm{ST}}$ values based on **a** 700 k data except for the indicine reference breeds which are based on the 35 k data; and **b** for the BAIF indigenous breeds only based on the 700 k data

Figure 8a shows a heat map based on the ${F}_{\mathrm{ST}}$ values between all reference and indigenous breeds. ${F}_{\mathrm{ST}}$ measures the degree of genetic difference between breeds, with high ${F}_{\mathrm{ST}}$ values (colored in red in the heat maps) indicating greater genetic distances. Where possible, pair-wise ${F}_{\mathrm{ST}}$ values were based on the 700 k data to allow the capture of as much genetic variation as possible. A hierarchical clustering was used to order the breeds in the matrix. As expected, the exotic dairy breeds show relatively small genetic distances with each other, and this is observed within the indicine reference breeds and the Indian indigenous breeds in our dataset, which are genetically distant from the *Bos taurus* exotic dairy breeds. It also shows that genetic distances between the indicine reference and our Indian indigenous breeds are substantially shorter than those between the exotic dairy breeds, which confirms the results reported by Nayee et al. [21].

Figure 8b is a heat map for ${F}_{\mathrm{ST}}$ and hierarchical clustering using only the Indian indigenous breeds sampled for our study to highlight finer details. The results are similar to the patterns obtained in the PC and admixture analyses. Figure 6b shows that the Red Sindhi animals are genetically distinct from all the other indigenous breeds, followed in decreasing order by Gir, Sahiwal and Tharparkar, which confirms a similar pattern reported by Nayee et al. [21]. The non-descript animals are the least genetically distant from all other breeds, closely followed by the Krishna Valley breed.

Figure 1d shows the maximum likelihood tree among all the reference and indigenous breeds obtained by TreeMix using the 35 k data. The tree clearly separates *Bos taurus* from *Bos indicus* breeds and also shows that the genetic distances are greater between breeds within *Bos taurus* than within *Bos indicus* [9, 26, 42]*.* The relationships between the indicine reference breeds and our Indian indigenous breeds are associated with relatively high uncertainties because of the small sample sizes of most of the reference breeds coupled with the relatively low levels of genetic diversity observed for the *Bos indicus* breeds. Nevertheless, the indigenous Gir and the reference Gir (from Brazil) samples that both include 20 animals, cluster together. Similarly, the reference Nelore (from Brazil) and reference Ongole (from India) samples, each with 20 animals, also cluster together.

Figure 1e shows the maximum likelihood tree applied only to the Indian indigenous breeds sampled for our study and using the 700 k data. These results are based on substantially more animals per breed than the reference samples. The tree shows that Red Sindhi is the most distant breed from all the other breeds, which confirms the results of Gajjar et al. [42]. As noted earlier, this may reflect that the Red Sindhi animals were sampled from several larger herds with a resulting high degree of relationship within the sample, which may not be representative of the breed as a whole. Tharparkar, Gir, Hariana, and Sahiwal, which are all dairy or dual purpose dairy-draught breeds from the North West of India, cluster together. Conversely, the four breeds sampled from Maharashtra—Dangi, Gaolao, Khillar and Red Kandhari—are not more closely related to each other than other breeds that are not from North West India. The non-descript population seems to have a common ancestor with Red Sindhi, however, this might also be an artefact due to the higher diversity found for Red Sindhi rather than a true evolutionary history. The results also indicate migration from Dangi and a common ancestor for Khillar and Hallikar to the non-descript population. This multi-breed background of the non-descript population partially confirms the above-described results based on admixture and f3 statistics.

The f4 statistic was used to test whether two clusters of four populations (A,B;C,D) have a significant gene flow between them. Most often, the Hallikar breed closely followed by the non-descript population are included in the clusters that show a significant gene flow, whereas the Gaolao and Malnad Gidda breeds are less commonly included in clusters with gene flow. Gene flow is mostly observed between the non-descript population and the Dangi breed, i.e. 122 times in 1498 clusters, followed by Khillar and Hallikar (108 times), Hallikar and Krishna Valley (83 times), and Gir and Red Kandhari (77 times), which is in concordance with the maximum likelihood tree and the identified migration edges (see Additional file 10).

### Effective population sizes

**Fig. 9:**
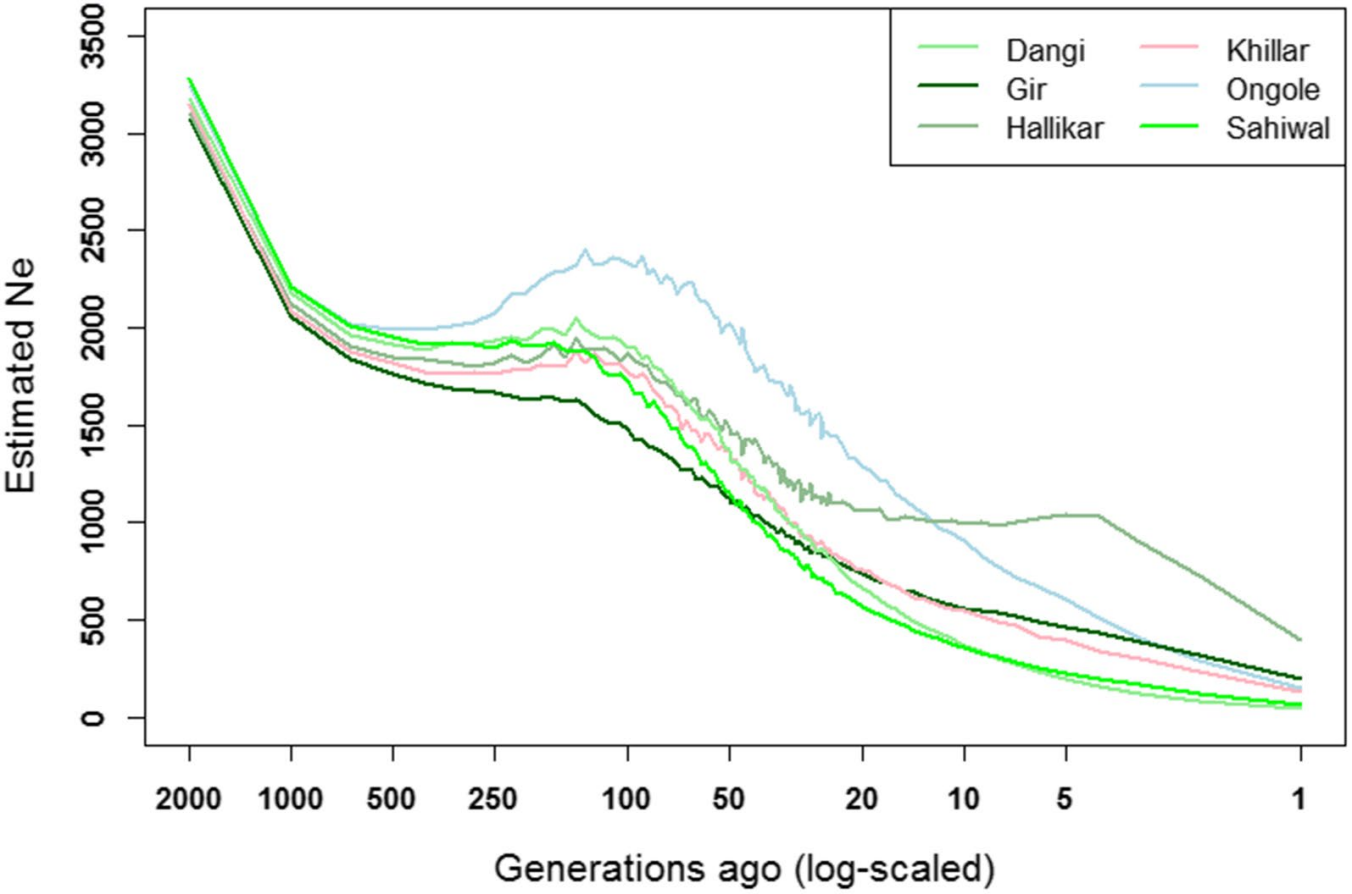
Change in estimated ${N}_{e}$ over time for six indigenous breeds (N > 20)

Figure 9 shows the evolution of the estimated ${N}_{e}$ over time for the Indian indigenous breeds based on the LD between SNPs separated by 0 to 50 Mb. To increase the reliability of the results, only the breeds with more than 20 animals after removing the related individuals are shown. Additional file 10 Figure S6 shows the results for the other breeds for which ${N}_{e}$ is likely underestimated due to their small sample size, and Additional file 12 Figure S7 shows the decay of LD with increasing distance between SNPs. All the breeds start with a ${N}_{e}$ larger than 3000 and larger than 2000 at 2000 and 1000 generations ago, respectively. Assuming that the average generation interval in cattle is about 5 years, these numbers describe the ${N}_{e}$ around the time of the first domestication of *Bos indicus* cattle, i.e. somewhere between 10,000 and 5000 BP. The fact that similar estimates are obtained for the different breeds is consistent with the various Indian indigenous breeds being derived from one large cattle population. All breeds show a levelling or even increasing ${N}_{e}$ between 500 and 100 generations ago, and ${N}_{e}$ begins to strongly deviate between breeds, which suggest that populations went through a period of expansion in numbers during this period. Subsequently, ${N}_{e}$ started to decline, perhaps as breed characteristics became more clearly defined and the farmer-breeders, consciously or unconsciously, became more selective about which animals to breed from to maintain breed characteristics. For the Hallikar breed, it seems that, during the period between 25 and 5 generations ago, ${N}_{e}$ remained relatively stable at 1000 individuals. During this period in the nineteenth century, Hallikar was classified as one of the three breeds from which the Amrit Mahal breed originated, a breed that received great attention as drought animals for army use. In addition to this reason, which could already have led to an increase in population size, the three independent Amrit Mahal varieties, Hallikar, Hagalvadi, and Chitaldroog, were merged into one registered breed for economic reasons [46], and thus the Hallikar breed as known today might have benefitted from this reverse bottleneck. Ongole is the breed that seems to have expanded the most post-domestication, possibly for the same reasons that they are still favored today, i.e. fast growth, heat resistance and disease resistance. In more recent times, i.e. over the past 50 generation, the Gir breed shows the smallest relative decline in ${N}_{e}$, whereas Dangi and Sahiwal cattle show a relatively high decline.

Table 2 shows the estimated ${N}_{e}$ for all the breeds at 1, 5 and 2000 generations before present. At one generation ago, all the breeds have a relatively small ${N}_{e}$, with the largest ${N}_{e}$ found for Hallikar (${N}_{e}$ = 399), followed by Gir (${N}_{e}$ = 197), and Ongole (${N}_{e}$ = 151). However, these ${N}_{e}$ need to be interpreted with caution, since the number of SNPs per bin was small in this analysis. As previously shown, the low ${F}_{\mathrm{IS}}$ and high ${F}_{\mathrm{ROH}}$ values found for the Indian indigenous breeds from our study corroborate the current small ${N}_{e}$. In recent generations, ${N}_{e}$ has decreased dramatically, i.e. for the breeds in Fig. 8${N}_{e}$ are currently about 25 to 50% the size that they were five generations ago. In many cases, ${N}_{e}$ approaches values that are found for intensively bred *Bos taurus* breeds [47, 48]. Thus, care must be taken in the ongoing genetic improvement programs for several indigenous breeds. Indeed, such programs could rapidly drive ${N}_{e}$ even further down and drive up inbreeding rates unless genetic selection and mating processes are put in place that aim at balancing between maintaining ${N}_{e}$ and increasing genetic merit.

## Conclusions

The main result from this investigation is the surprisingly low genetic diversity observed among the indigenous breeds that were sampled across different states and agro-environmental regions of India. This observation still held when SNPs with a low MAF were excluded to minimize potential effects of ascertainment bias of the SNP assay. A possible explanation of these relatively low levels of genetic diversity is that the effective population sizes of these Indian indigenous breeds have been large since domestication. Thus, the Indian indigenous breeds may have maintained allele frequencies, which have been less affected by genetic drift since domestication and more closely reflecting those in the original population before the differentiation into the current breeds. Future developments in SNP assay designs to include a more representative sample of *Bos indicus* breeds might allow the capture of more of the genetic diversity between these breeds.

## Supplementary Information

**Additional file 1. Table S1.** Exotic and indicine reference breeds and India *Bos indicus* breeds sampled for this study.

**Additional file 2: Material and Methods S1.** Genomic relationship matrix calculations. **S2.** Calculation of allele frequency error variances. **S3.** Linkage disequilibrium calculations.

**Additional file 3: Figure S1.** PC3 to 6 obtained with up to 20 animals per pure BAIF indigenous breed.

**Additional file 4: Figure S2.** Allele frequencies with 35 k SNPs.

**Additional file 5: Figure S3.** Allele frequencies with 700 k SNPs.

**Additional file 6: Table S2.** Correlations of SNP allele frequencies between exotic dairy breeds based on the 700 k data.

**Additional file 7. Figure S4. **Estimated breed proportions for the BAIF indigenous samples from an unsupervised admixture analysis with K ranging from 2 to 11.

**Additional file 8. Figure S5.** Cross-validation error for the unsupervised admixture analyses with K ranging from 2 to 11.

**Additional file 9.** f3 statistics from TreeMix for the BAIF indigenous samples.

**Additional file 10.** f4 statistics from TreeMix for the BAIF indigenous samples.

**Additional file 11: Figure S6.** Change in the estimated Ne over time for eight indigenous breeds (N < 20).

**Additional file 12: Figure S7.** Decay of the linkage disequilibrium with increasing distance between SNPs for **a** six indigenous breeds (N > 2f0) and **b** eight indigenous breeds (N < 20).

## Data Availability

The data generated specifically for this study are available from M. Swaminathan mswami@baif.org.in on reasonable request. Reference data are available from public and private databases as described in the paper.
