# Supplementary material for: Genetic diversity and effective population sizes of thirteen Indian cattle breeds
Source: Genet Sel Evol. 2021 Jun 1;53:47. doi: 10.1186/s12711-021-00640-3 (PMC8170732; doi:10.1186/s12711-021-00640-3)
Supplement: Supplementary file 1 — Additional file 1. Table S1. Exotic and indicine reference breeds and India Bos indicus breeds sampled for this study. [file 12711_2021_640_MOESM1_ESM.docx]

**Additional file 1 Table S1** Exotic and indicine reference breeds and India Bos indicus breeds sampled for this study

| Breed | N | Sampling Location | Source | # SNPs | Excluded* |
| --- | --- | --- | --- | --- | --- |
| Holstein | 20 |  | HapMap | 777k |  |
| Jersey | 20 |  | HapMap | 777k |  |
| Ayrshire | 20 |  | Canadian Dairy Network (CDN) | 777k |  |
| British Friesian | 20 |  | SRUC | 777k |  |
| Guernsey | 20 |  | HapMap | 777k |  |
| Brown Swiss | 20 | USA, America | Decker et al. 2014 (26) | 777k |  |
| Achai | 12 |  | Decker et al. 2014 (26) | 50k | 12 |
| Bhagnani | 10 | Kaochi, Kalat, and Baluchistan, Pakistan | Decker et al. 2014 (26) | 50k | 3 |
| Cholistani | 11 | Punjab, Pakistan | Decker et al. 2014 (26) | 50k |  |
| Dajal | 10 | Punjab, Pakistan | Decker et al. 2014 (26) | 50k | 1 |
| Dhanni | 12 | Punjab, Pakistan | Decker et al. 2014 (26) | 50k |  |
| Gabrali | 10 | Khyber Pakhtun Khwa, Pakistan | Decker et al. 2014 (26) | 50k | 10 |
| Gir | 20 | Brazil | HapMap | 777k |  |
| Guzerat | 3+8 | Guzarat, India | HapMap+Decker et al. 2014 (26) | 777k + 50k |  |
| Hariana | 10 | Hariana, India | Decker et al. 2014 (26) | 50k |  |
| Hissar | 10 | Punjab, Pakistan | Decker et al. 2014 (26) | 50k |  |
| Kankraj | 10 | North Gujerat, India | Decker et al. 2014 (26) | 50k |  |
| Lohani | 10 | Northwest Pakistan | Decker et al. 2014 (26) | 50k | 2 |
| Nelore | 20 | Brazil | HapMap | 777k | 2 |
| Ongole | 20 | Andhra Pradesh, India | Decker et al. 2014 (26) | 50k | 9 |
| Red Sindhi | 10 | Sindhi, Pakistan | Decker et al. 2014 (26) | 50k | 2 |
| Rojhan | 10 | Punjab, Pakistan | Decker et al. 2014 (26) | 50k |  |
| Sahiwal | 17 | Punjab, Pakistan | Decker et al. 2014 (26) | 50k |  |
| Tharparkar | 12 | Southeast Sindhi, Pakistan | Decker et al. 2014 (26) | 50k | 3 |
| Dangi | 68 | Maharashtra, India | This study | 777k | 3 |
| Gaoloa | 20 | Maharashtra, India | This study | 777k | 1 |
| Gir | 121 | Gujarat, India | This study | 777k | 3 |
| Hallikar | 28 | Karnataka, India | This study | 777k | 2 |
| Haryana | 17 | Haryana, India | This study | 777k | 4 |
| Khillar | 25 | Maharashtra, India | This study | 777k | 1 |
| Krishna Valley | 22 | Karnataka, India | This study | 777k | 5 |
| Red Kandhari | 35 | Maharashtra, India | This study | 777k |  |
| Malnad Gidda | 19 | Karnataka, India | This study | 777k | 5 |
| Ongole | 50 | Andhra Pradesh, India | This study | 777k |  |
| Rathi | 1 | Rajasthan, India | This study | 777k |  |
| Red Sindhi | 63 | Odisha, India | This study | 777k | 20 |
| Sahiwal | 140 | Punjab, India | This study | 777k | 36 |
| Tharparkar | 48 | Rajasthan, India | This study | 777k | 3 |
| Vechur | 1 | Kerala, India | This study | 777k |  |
| Non-descript (ND) | 43 |  | This study | 777k | 27 |

*Exclusion of individuals based on preliminary Admixture results where more than 1% of exotic breed ancestry was found; an exception was made for Guzerat which clustered tightly together in principal component analysis but all animals had slightly more than 1% exotic breed proportion
