# Supplementary material for: Genetic diversity and effective population sizes of thirteen Indian cattle breeds
Source: Genet Sel Evol. 2021 Jun 1;53:47. doi: 10.1186/s12711-021-00640-3 (PMC8170732; doi:10.1186/s12711-021-00640-3)
Supplement: Supplementary file 2 — Additional file 2: Material and Methods S1. Genomic relationship matrix calculations. S2. Calculation of allele frequency error variances. S3. Linkage disequilibrium calculations. [file 12711_2021_640_MOESM2_ESM.docx]

Additional file 2

Genetic Diversity and Effective Population Sizes of Thirteen Indian Cattle Breeds

**Material and Methods S1**

Principal components (PC) were calculated based on the genomic relationship matrix (GRM) which was derived with two approaches. The first approach constructed the GRM according to **Van Raden (28)**. Missing genotypes were replaced by average genotypes across all animals.

1. $\mathbf{GRM}=\mathbf{ZZ}’/(2*\sum p_{l}*(1-p_{l}))$,

where $\mathbf{Z}$ is the centered genotype matrix and $p$ is the allele frequency at locus $l$. Matrix $\mathbf{Z}$ was constructed by subtracting from the genotype matrix $\mathbf{M}$ the $\mathbf{P}$ vector, which equaled 2*($p$-0.5). The centering of $\mathbf{Z}$ was achieved by subtracting -1 from $\mathbf{M}$.

The second approach constructed the GRM according to **Yang et al. (29)** and used a standardized genotype matrix **W**.

$$\mathbf{W}=(\mathbf{M-}2p_{l}\mathbf{)}/\sqrt{2p(1-p_{l})}$$

where **M** is the genotype matrix containing allele counts with missing genotypes replaced by average genotypes across animals, and p is the allele frequency at locus *l*.

$\mathbf{GRM}=\mathbf{WW}’/N$,

where $\mathbf{W}$ is the standardized genotype matrix and N is the total number of SNPs.

Note that the GRMs are constructed across multiple breeds which skews values of diagonal and off-diagonal elements for some breeds, especially where the breed has different allele frequencies to the other breeds **(25)**. Therefore, we constructed the GRMs in four different ways, using: 1) all reference breeds plus the Indian indigenous breeds sampled here, 2) only the exotic reference breeds, 3) only the indicine reference breeds, and 4) only the Indian indigenous breeds. Each GRM was chosen for the appropriate follow-up analysis.

**Material and Methods S2**

The error variances V_e1_ and V_e2_ were estimated as the average across all loci as *p*(1-*p*)/2n, where n is the number of animals in the given breed and *p* is the meta-population value of *p* for each SNP. V_p_ was not corrected for the sampling error of *p*, which in all cases was less than 1% of the estimate of V_p_.

**Material and Methods S3**

Linkage disequilibrium was estimated as the *r*^2^ across animals per breed between all pairs of alleles of all SNP-pairs in a given panel of SNP **(38)**, using VCFtools **(39)**:

$$r^{2}=\frac{D^{2}}{f\left( A \right)\times f(a)\times f(B)\times f(b)}$$

where,

$$D=f\left( AB \right)-f\left( A \right)f(B)$$

and *f(AB)* is the estimated frequency of haplotype AB using the observed genotype frequency **(40)** and assuming Hardy Weinberg equilibrium; while *f(A)*, *f(a)*, *f(B)* and *f(b)* are observed frequencies of alleles *A*, *a*, *B*, and *b*, respectively.
