# Supplementary figures and images for: Genetic diversity and effective population sizes of thirteen Indian cattle breeds
Source: Genet Sel Evol. 2021 Jun 1;53:47. doi: 10.1186/s12711-021-00640-3 (PMC8170732; doi:10.1186/s12711-021-00640-3)

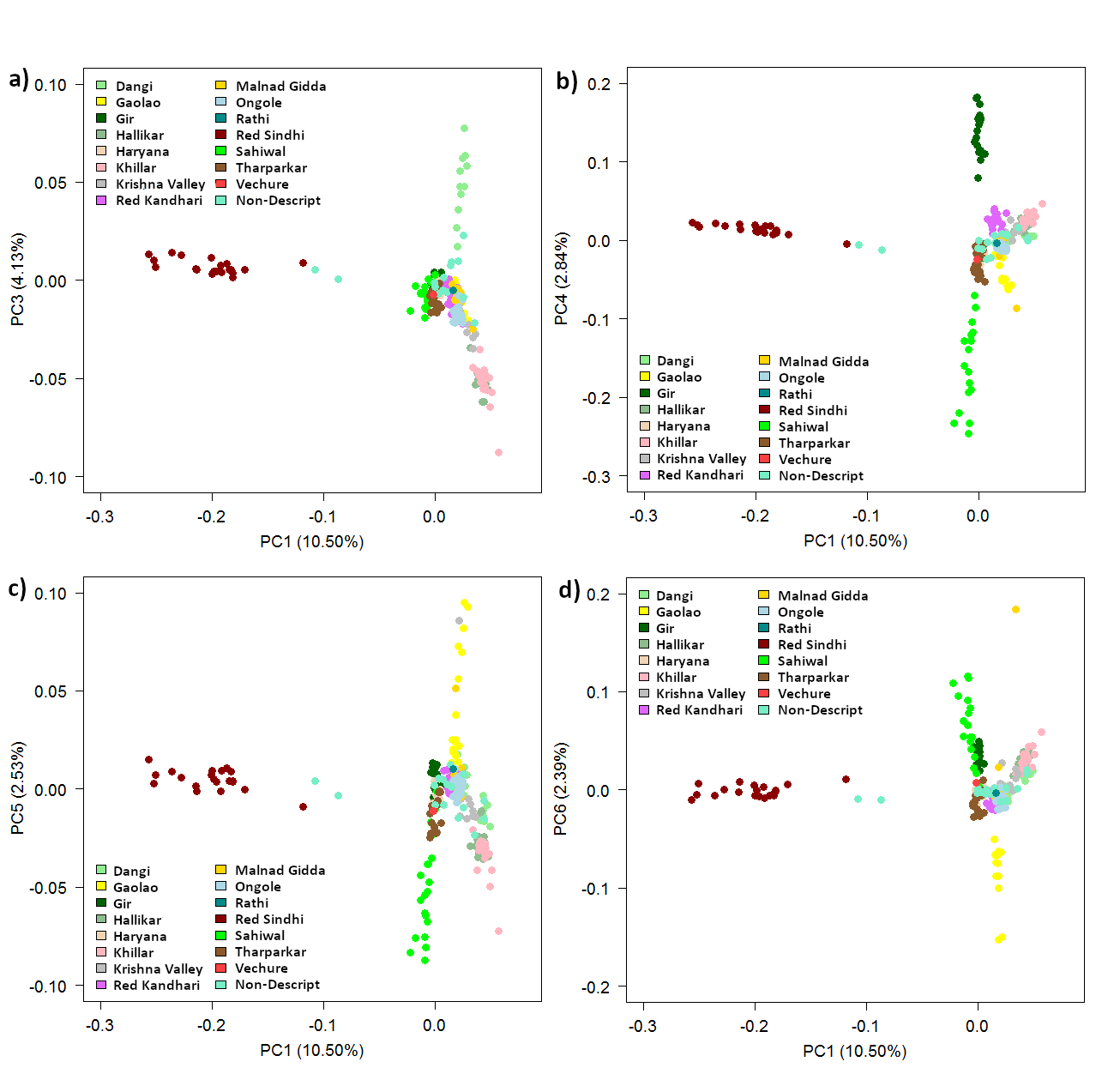

Supplement: Supplementary file 3 — Additional file 3: Figure S1. PC3 to 6 obtained with up to 20 animals per pure BAIF indigenous breed. [file 12711_2021_640_MOESM3_ESM.tiff]

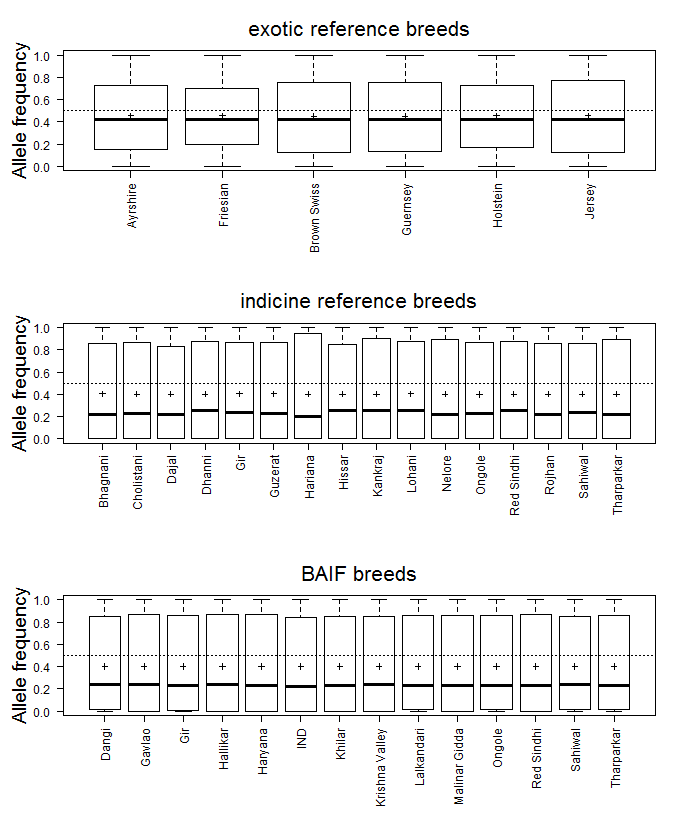

Supplement: Supplementary file 4 — Additional file 4: Figure S2. Allele frequencies with 35 k SNPs. [file 12711_2021_640_MOESM4_ESM.tiff]

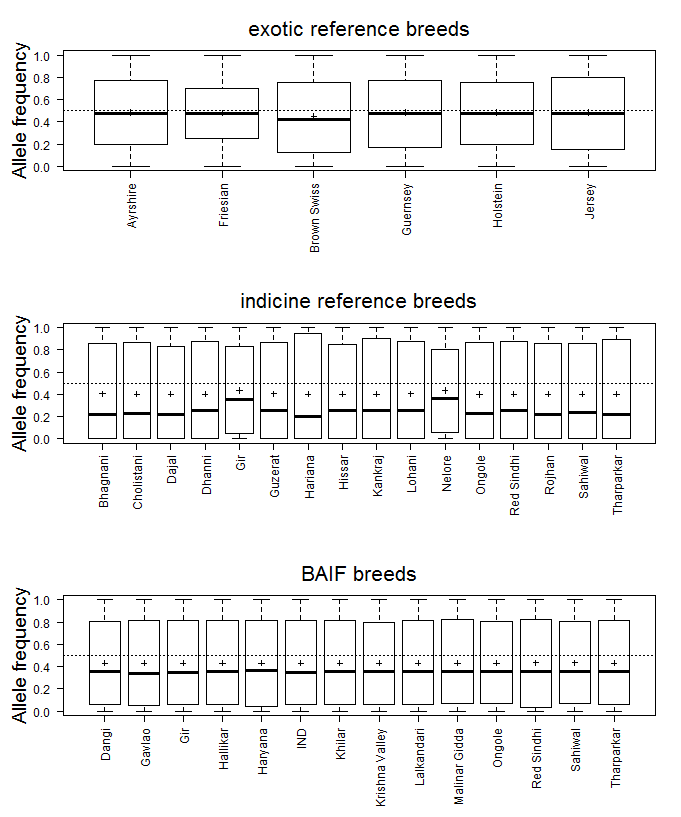

Supplement: Supplementary file 5 — Additional file 5: Figure S3. Allele frequencies with 700 k SNPs. [file 12711_2021_640_MOESM5_ESM.tiff]

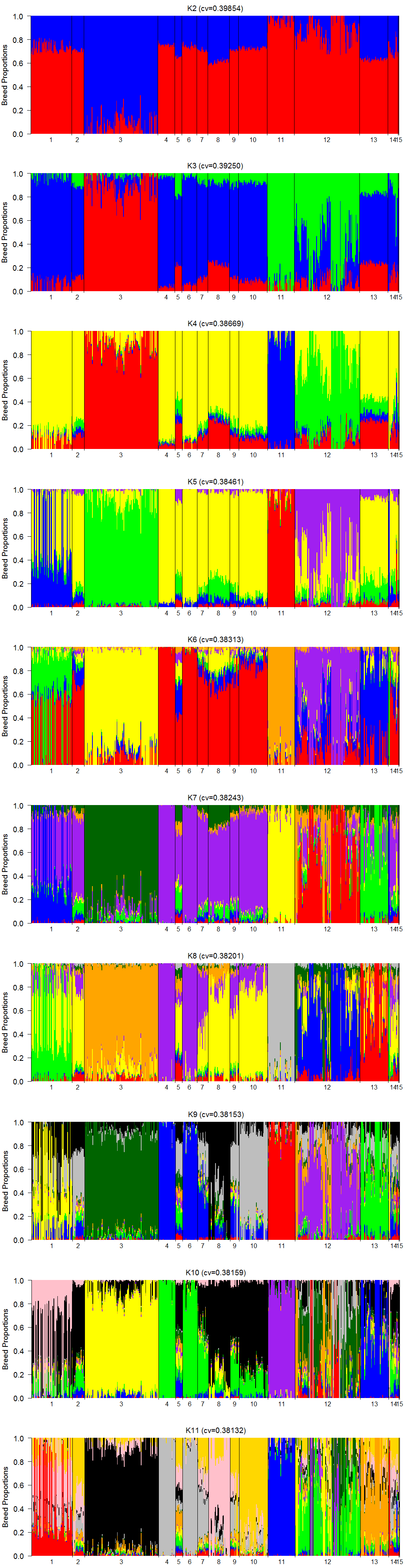

Supplement: Supplementary file 7 — Additional file 7. Figure S4. Estimated breed proportions for the BAIF indigenous samples from an unsupervised admixture analysis with K ranging from 2 to 11. [file 12711_2021_640_MOESM7_ESM.tiff]

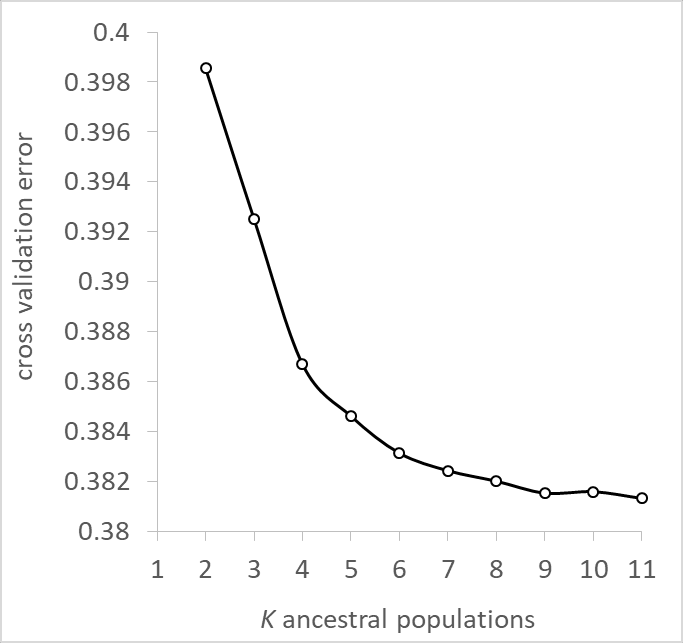

Supplement: Supplementary file 8 — Additional file 8. Figure S5. Cross-validation error for the unsupervised admixture analyses with K ranging from 2 to 11. [file 12711_2021_640_MOESM8_ESM.tiff]

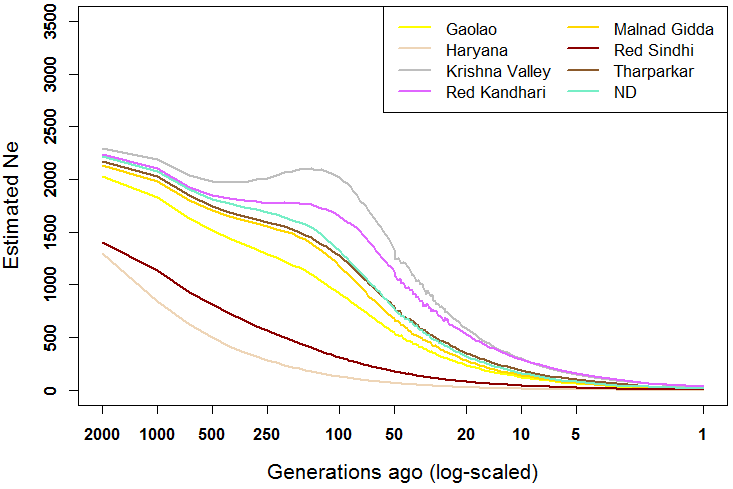

Supplement: Supplementary file 11 — Additional file 11: Figure S6. Change in the estimated Ne over time for eight indigenous breeds (N < 20). [file 12711_2021_640_MOESM11_ESM.tiff]

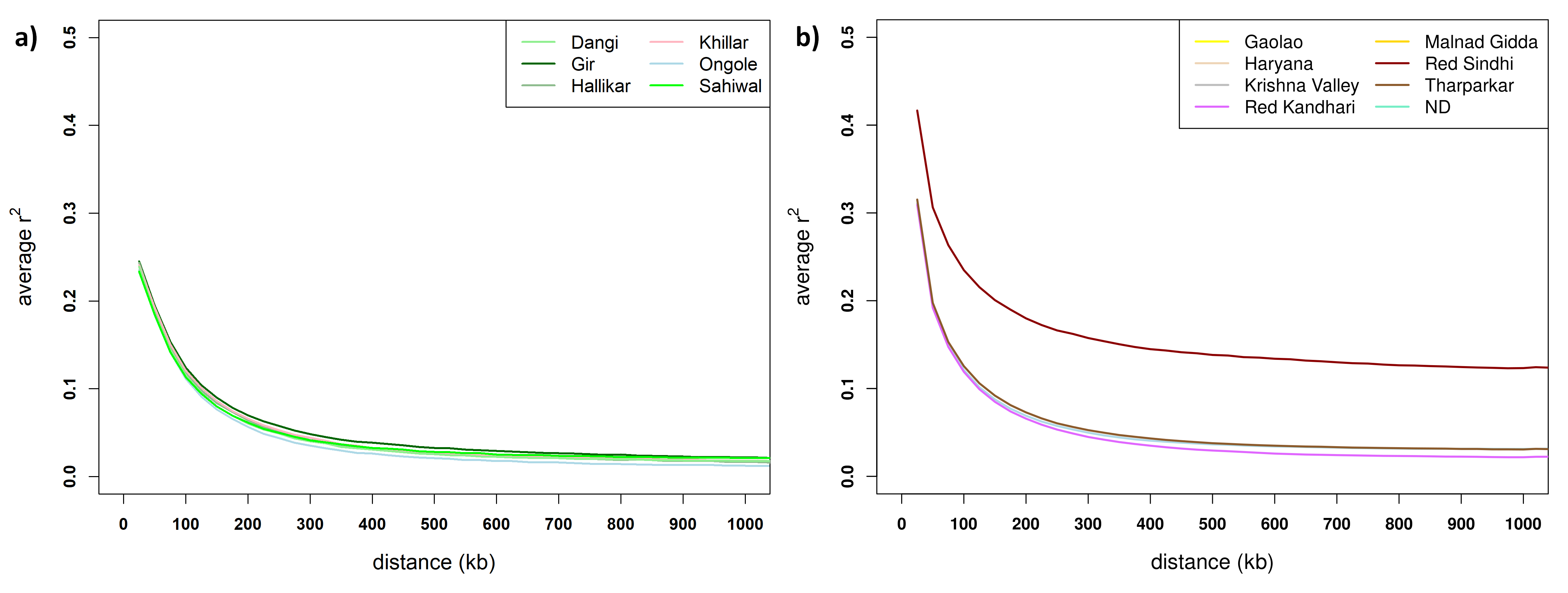

Supplement: Supplementary file 12 — Additional file 12: Figure S7. Decay of the linkage disequilibrium with increasing distance between SNPs for a six indigenous breeds (N > 2f0) and b eight indigenous breeds (N < 20). [file 12711_2021_640_MOESM12_ESM.tiff]
