# Supplementary material for: Genetic diversity and effective population sizes of thirteen Indian cattle breeds
Source: Genet Sel Evol. 2021 Jun 1;53:47. doi: 10.1186/s12711-021-00640-3 (PMC8170732; doi:10.1186/s12711-021-00640-3)
Supplement: Supplementary file 6 — Additional file 6: Table S2. Correlations of SNP allele frequencies between exotic dairy breeds based on the 700 k data. [file 12711_2021_640_MOESM6_ESM.docx]

**Additional file 6 Table S2** Correlations of SNP allele frequencies between exotic dairy breeds based on 700k data

|  | Ayrshire | Friesian | Guernsey | Holstein | Jersey | Brown Swiss |
| --- | --- | --- | --- | --- | --- | --- |
| Ayrshire |  | 0.774 | 0.708 | 0.724 | 0.683 | 0.703 |
| Friesian | 0.713 |  | 0.751 | 0.798 | 0.719 | 0.746 |
| Guernsey | 0.640 | 0.683 |  | 0.690 | 0.718 | 0.712 |
| Holstein | 0.659 | 0.744 | 0.614 |  | 0.662 | 0.685 |
| Jersey | 0.611 | 0.645 | 0.645 | 0.587 |  | 0.688 |
| Brown Swiss | 0.627 | 0.672 | 0.631 | 0.605 | 0.604 |  |
| **N** | **20** | **20** | **20** | **20** | **20** | **20** |

*Above diagonal: using all SNP; Below diagonal: using SNP with MAF > 0.05.
